# DELTEX E3 ligases ubiquitylate ADP-ribosyl modification on nucleic acids

**DOI:** 10.1093/nar/gkad1119

**Published:** 2023-11-24

**Authors:** Kang Zhu, Marcin J Suskiewicz, Chatrin Chatrin, Øyvind Strømland, Bryan W Dorsey, Vincent Aucagne, Dragana Ahel, Ivan Ahel

**Affiliations:** Sir William Dunn School of Pathology, University of Oxford, Oxford, UK; Centre de Biophysique Moléculaire, CNRS UPR 4301, Orléans, France; Sir William Dunn School of Pathology, University of Oxford, Oxford, UK; Sir William Dunn School of Pathology, University of Oxford, Oxford, UK; Department of Biomedicine, University of Bergen, Bergen, Norway; Ribon Therapeutics, 35 Cambridgepark Dr., Suite 300, Cambridge MA 02140, USA; Centre de Biophysique Moléculaire, CNRS UPR 4301, Orléans, France; Sir William Dunn School of Pathology, University of Oxford, Oxford, UK; Sir William Dunn School of Pathology, University of Oxford, Oxford, UK

## Abstract

Although ubiquitylation had traditionally been considered limited to proteins, the discovery of non-proteinaceous substrates (e.g. lipopolysaccharides and adenosine diphosphate ribose (ADPr)) challenged this perspective. Our recent study showed that DTX2 E3 ligase efficiently ubiquitylates ADPr. Here, we show that the ADPr ubiquitylation activity is also present in another DELTEX family member, DTX3L, analysed both as an isolated catalytic fragment and the full-length PARP9:DTX3L complex, suggesting that it is a general feature of the DELTEX family. Since structural predictions show that DTX3L possesses single-stranded nucleic acids binding ability and given the fact that nucleic acids have recently emerged as substrates for ADP-ribosylation, we asked whether DELTEX E3s might catalyse ubiquitylation of an ADPr moiety linked to nucleic acids. Indeed, we show that DTX3L and DTX2 are capable of ubiquitylating ADP-ribosylated DNA and RNA synthesized by PARPs, including PARP14. Furthermore, we demonstrate that the Ub-ADPr-nucleic acids conjugate can be reversed by two groups of hydrolases, which remove either the whole adduct (e.g. SARS-CoV-2 Mac1 or PARP14 macrodomain 1) or just the Ub (e.g. SARS-CoV-2 PLpro). Overall, this study reveals ADPr ubiquitylation as a general function of the DELTEX family E3s and presents the evidence of reversible ubiquitylation of ADP-ribosylated nucleic acids.

## Graphical Abstract

**Graphical Abstract:**
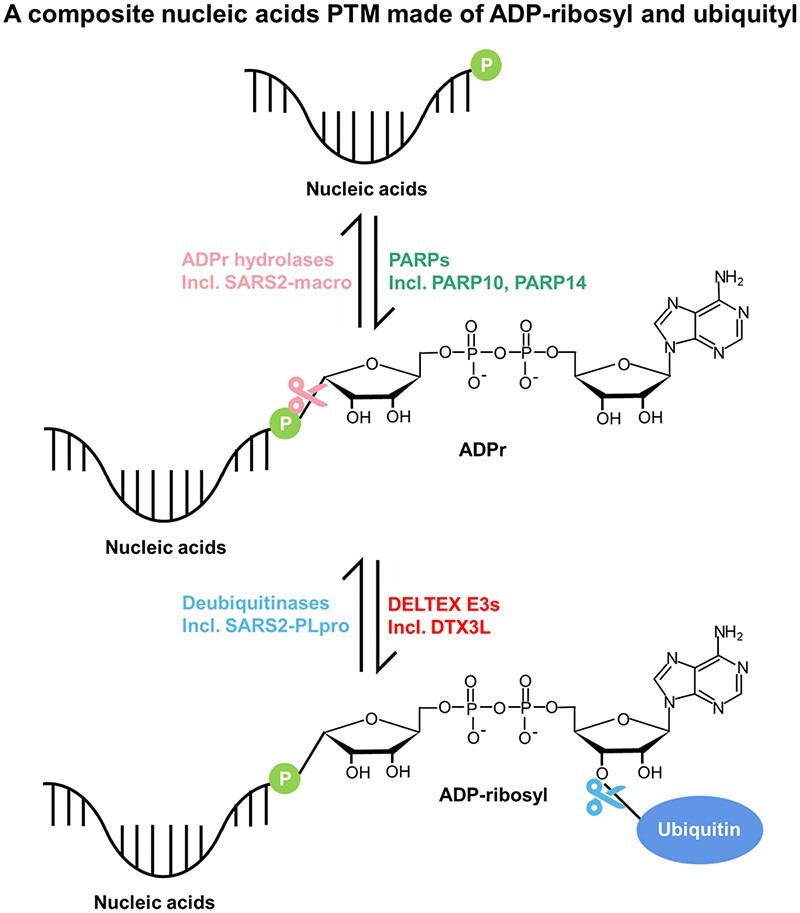

## Introduction

As one of the most investigated posttranslational modifications (PTMs), ubiquitylation (or ubiquitination) refers to the attachment of one or more ubiquitin (Ub) units to protein substrates, which regulates protein activity, stability, localization and interactome (1,2). Ubiquitylation is a highly orchestrated process catalysed by three classes of enzymes: Ub-activating enzymes (E1s), Ub-conjugating enzymes (E2s) and Ub protein ligases (E3s). Ub is first activated in an ATP-dependent manner by an E1 enzyme and subsequently transferred to the catalytic cysteine residue of an E2 enzyme to form the E2∼Ub thioester, followed by Ub conjugation to a protein substrate catalysed by an E3 enzyme (3,4). Ub is mainly attached to protein substrates via an isopeptide bond between the substrate's lysine/N-terminal amino group and the C-terminal glycine residue of Ub (Gly^76^). Moreover, some E3 ligases can conjugate Ub to the hydroxyl groups of threonine and serine residues (5,6) or a thiol group of a cysteine residue (7). Ubiquitylation, like other PTMs, is a highly dynamic and reversible modification and is removed by deubiquitinases (DUBs) (8).

Proteins have traditionally been considered the sole substrates for ubiquitylation, but there is accumulating evidence that non-proteinaceous ubiquitylation substrates also exist. The first discovered substrate of the latter kind is the bacterial lipopolysaccharide (LPS), which is ubiquitylated by the host E3 ligase RNF213 during *Salmonella* infection (9). More recently, a linear ubiquitin chain assembly complex (LUBAC) component, the E3 ligase HOIL-1, was shown to catalyse sugar ubiquitylation, specifically on sugar hydroxyl groups (10). Furthermore, phosphatidylethanolamine (PE) (a phospholipid) is ubiquitylated on its head group via an amide bond by an enzymatic cascade composed of Uba1 (E1), Ubc4/5 (E2) and Tul1 (E3) in yeast (11). In addition, our previous study showed, using a catalytic fragment of DTX2 as a representative, that the DELTEX family E3 ligases are capable of ubiquitylating a specific hydroxyl group within adenosine diphosphate (ADP) ribose (ADP-ribose or ADPr) or nicotinamide adenine dinucleotide (NAD^+^) *in vitro* (12) (see below). These newly discovered non-proteinaceous substrates broaden our understanding of ubiquitylation and provide new research avenues to explore.

In addition to free ADPr/NAD^+^, DELTEX E3 ligases can also ubiquitylate an ADPr moiety that is covalently linked to a protein substrate as a result of a prior ADP-ribosylation reaction (12). ADP-ribosylation is an abundant and rapid cellular PTM, which transfers ADPr using NAD^+^ as a cofactor, onto target substrates. ADP-ribosylation is catalysed by enzymes known as ADP-ribosyltransferases (ARTs), which include the PARP family (13–16). PARP1, the founding member of this protein family, is well known for its crucial roles in the DNA damage response (DDR) pathway (17). Outside of a role in DDR, ADP-ribosylation regulates a variety of other cellular processes including chromatin relaxation, RNA biogenesis, stress granule formation, immune responses and many more (14,18). During the ADP-ribosylation reaction, a single or multiple ADPr moieties can be conjugated to substrates in a process known as mono-ADP-ribosylation or poly(ADP-ribosyl)ation, respectively, in the latter case resulting in the formation of a poly(ADPr) (PAR) chain. ADP-ribosylation is a reversible process, with several ADPr hydrolases catalysing ADPr removal, including MacroD1/2, ARH1/3, TARG1 and PARG (19). Among these enzymes, only PARG is unable to reverse the last ADPr moiety attached directly to the substrates. PARG instead efficiently cleaves the inter-ADPr bonds within a PAR chain (20). The other ADPr hydrolases complement PARG’s function by removing the last ADPr attached to the substrates, with different enzymes showing varying specificities for particular types of ADPr-substrate linkages. For example, MacroD1/2 and TARG1 mostly reverse the Asp/Glu-linked ADP-ribosylation (21–24), while ARH1 and ARH3 cleave Arg- and Ser-ADP-ribosylation, respectively (25–27), where the latter is the major signal during DNA damage response produced by PARP1 or PARP2 in complex with the accessory factor HPF1 (28,29). Regarding PAR chain hydrolysis, in addition to PARG as the main chain-specific hydrolase, ARH3 also shows some, lower, PAR trimming activity (25,30,31). ADP-ribosylation is not exclusive to proteins, but also occurs on nucleic acids (32–35). Pierisin toxins from cabbage butterfly larvae was the first enzyme described to irreversibly ADP-ribosylate DNA on guanine (36), followed by the discovery of the bacterial DarTG toxin-antitoxin systems in which different DarT toxin enzymes specifically modify thymidine or guanine residues in single-stranded DNA (in a sequence specific manner) (35,37,38). Furthermore, in the past several years, it has been shown that PARP1, PARP2, and PARP3 modify double-stranded DNA on phosphorylated termini, such as those found at sites of DNA breaks (39–41). Moreover, several PARPs including PARP10 and PARP14 ADP-ribosylate phosphorylated single-stranded RNA (ssRNA) and/or single-stranded DNA (ssDNA) ends (42,43). It has been shown that PARG, ARH3, TARG1 and MacroD1/2 can all reverse the ADP-ribosylation on the phosphate moieties of ssDNA or ssRNA ends (39,42).

Human DELTEX family E3s are homologues of the *Drosophila* Deltex protein, which plays important roles in Notch signaling that regulates cell-fate determination during development and maintains adult tissue homeostasis (44), indicating that DELTEX E3s might be involved in this pathway. Recent studies suggested that several DELTEX E3s are involved in DNA damage response (45–47). The DELTEX family is composed of five members, namely DTX1, DTX2, DTX3, DTX4 and DTX3L (48,49), of which the last-mentioned forms a tight complex with PARP9, a putative ADP-ribosyltransferase (45,50). The overexpression of the PARP9:DTX3L complex is observed in various cancers such as prostate and breast cancers, indicating its promoting role in the growth of cancer cells (45). PARP9:DTX3L was also suggested to function in DNA damage response, specifically in the non-homologous end joining (NHEJ) pathway (45). In addition, viral infection or interferon (IFN) treatment causes a rapid and strong upregulation of PARP9:DTX3L, which executes antiviral roles by contributing to the degradation of viral proteases and chromatin relaxation for interferon-stimulated gene (ISGs) expression (51).

**Figure 1.**
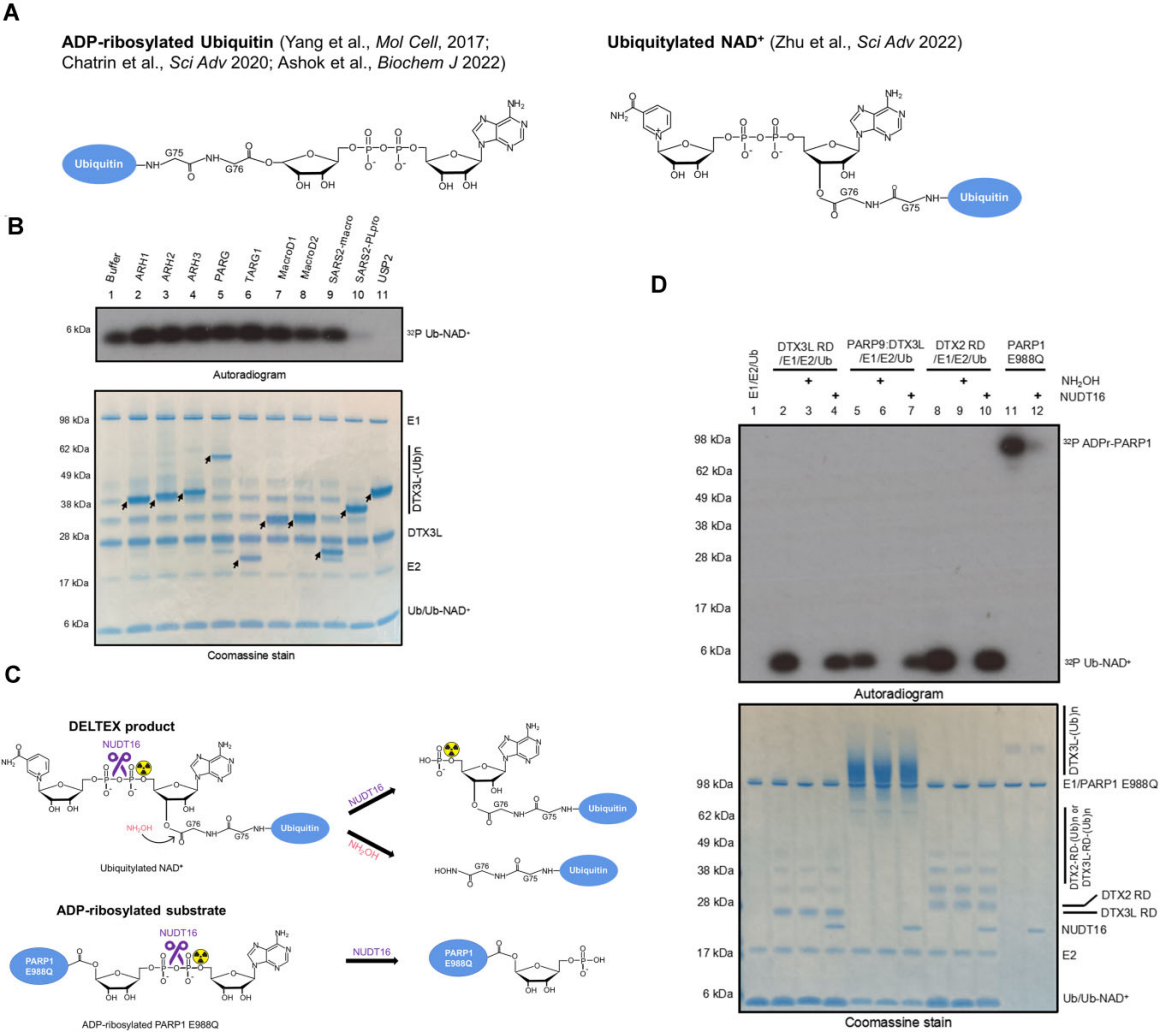
Biochemical characterization of PARP9:DTX3L catalysed NAD^+^ ubiquitylation. (**A**) Schematic diagrams showing the proposed DELTEX product in different studies. *Left panel*: ADP-ribosylated Ub; *Right panel*: ubiquitylated NAD^+^. (**B**) Hydrolysis of DTX3L RD catalysed Ub-NAD^+^. Following one reaction performed with DTX3L RD in (B), the indicated ADPr hydrolases and DUBs were added and further incubated. The samples were analyzed on an SDS-PAGE gel, which is then visualized by Coomassie staining and autoradiography. The arrows indicate various hydrolases. The arrows indicate various hydrolases. (**C**) Schematic diagram showing the NUDT16 cleavage specificity of ADP-ribosylated product and DELTEX product. NUDT16 treatment reverses radioactivity from ^32^P ADP-ribosylated PARP1 E988Q but not the ^32^P Ub-NAD^+^. (**D**) PARP9:DTX3L and DELTEX E3s ubiquitylate NAD^+^ on the adenine-proximal ribose. ^32^P Ub-NAD^+^ was generated by incubation of indicated E3s with E1, E2, ATP, Ub and NAD^+^ which is spiked with ^32^P NAD^+^, and next treated with NUDT16 or NH_2_OH. ^32^P ADP-ribosylated PARP1 E988Q was treated with NUDT16 to serve as control. The samples were analyzed on an SDS-PAGE gel, followed by Coomassie staining and autoradiography. NUDT16 removed radioactivity from PARP1 E988Q auto-modification but had no effect on the ^32^P Ub-NAD^+^ adduct, confirming that Ub is attached to the adenine-proximal ribose.

Biochemically, PARP9:DTX3L was first shown to catalyse a reaction between Ub and NAD^+^ with strict requirement for E1 and E2 enzymes and ATP *in vitro* (45). The product of the reaction was not directly identified, but it was provisionally annotated as C-terminally ADP-ribosylated Ub, i.e. Ub attached via its C-terminal carboxylate group to the C1″ atom of the adenosine-distal ribose of ADP-ribose (Figure 1A, *left panel*). Since PARP9 is thought to be inactive and did not show any auto-modification (52), the authors proposed that DTX3L might activate PARP9, enabling the proposed PARP9-mediated Ub ADP-ribosylation (45). Later, another study surprisingly showed that DTX3L but not PARP9 catalyses the reaction between Ub and NAD^+^, followed by the identification of adjacent RING and DELTEX C-terminal (DTC) domains (later jointly referred to as the RD tandem domain or RD region) as the minimal catalytically active fragment of DTX3L (53). The RD region is shared by all members of the DELTEX family, suggesting that they all might be able to catalyse the same reaction, whose chemical nature remained to be conclusively identified at that point. Therefore, in our previous study, we reconstituted the reaction catalysed by RD of DTX2 and characterized its product using various techniques. Using mass spectrometry, we proved that the real product, at least in the case of the DTX2 RD fragment, is not ADP-ribosylated Ub, but ubiquitylated NAD^+^ (Figure 1A). Furthermore, we showed that Ub is linked to NAD^+^ via an ester bond between the carboxylate group of Ub Gly^76^ and the 3′ hydroxyl group of adenosine-proximal ribose of NAD^+^ (Figure 1A, *right panel*). Finally, we showed that DTX2 RD ubiquitylates ADPr more efficiently than it modifies NAD^+^, and that it can also target an ADPr moiety installed on a protein or peptide (12). However, it remained theoretically possible, although in our view unlikely, that our results were not representative of the whole DELTEX family or of complete versions of these enzymes as opposed to their RD fragments. Such scenarios were invoked in a recent study and review (50,54), where the suggestion of ADP-ribosylated Ub as the correct product of the reaction between Ub and NAD^+^ catalysed by DTX3L catalytic fragment or PARP9:DTX3L was brought up once again.

In the current study, we revisited the question of the chemical nature of the product of DELTEX E3 ligases, this time focusing on the complete PARP9:DTX3L complex and the RD fragment of DTX3L. The analysis presented below confirmed that, contrary to previous suggestions, the results obtained with PARP9:DTX3L and DTX3L RD are consistent with previous observations for DTX2 RD in that, in all these cases, the product of the coupling between Ub and NAD*^+^* is ubiquitylated NAD^+^. Moreover, DTX3L RD, like DTX2 RD, can ubiquitylate free ADP-ribose and ADP-ribose installed on substrates. With regards to the last point, in light of recent identification of DNA and RNA as new substrates of ADP-ribosylation, we asked if DTX3L might also accept an ADPr moiety on ADP-ribosylated nucleic acids as a substrate. This was further motivated by our computational identification of previously unannotated putative ssDNA- or ssRNA-binding domains (KH and RRM) in DTX3L and recent identification of similar domains in PARP9 (43). Our EMSA assay result demonstrated the ssRNA binding ability of full-length DTX3L. Following these predictions and experimental validation, we demonstrated that DTX3L RD can ubiquitylate ADP-ribosylated ssDNA/RNA on the ADPr moiety *in vitro*, producing a potential dual Ub-ADPr- modification on nucleic acids. This process effectively links Ub to nucleic acids, a phenomenon that has not been reported so far, despite the recent advances in studying non-proteinaceous ubiquitylation. Overall, these results strengthen the notion that DELTEX E3 ligases generally ubiquitylate ADPr moieties and suggest novel substrates for ubiquitylation: ADP-ribosylated nucleic acids.

## Materials and methods

### Plasmids and protein purification

The PARP9 and DTX3L plasmids were generated by restriction digestion of pFastBac1 vector with EcoRI and NotI. The full-length gene of PARP9 (accession # NP_113646) with an N-terminal MBP tag and TEV cleavage site was inserted into the digested pFastBac1 vector. The same procedure was followed for the full-length gene of DTX3L (accession # NP_612144) with an N-terminal His_6_-tag and thrombin cleavage site. The MBP-TEV-PARP9 and His_6_-thrombin-DTX3L plasmid sequences were verified by Sanger sequencing.

WT and mutants of DTX2 RING-DTC and DTX3L RING-DTC were expressed and purified as previously described (12). Human ADPr hydrolases (PARG (55), ARH1-3 (25), MacroD1 (56,57), MacroD2 (23), and TARG1 (21)), SARS-CoV-2 Macro (12), SARS-CoV-2 PLpro (12), USP2 (12) and PARP14 WWE-CAT(43) were produced recombinantly before in our laboratory.

NUDT16 (ab103059) was purchased from Abcam; UBE1 (E-304-050), UBCH5A (E2-616-100), and recombinant Ub (WT and G76A; U-100H-10M and UM-G76A-100, respectively) were purchased from R&D Systems.

For PARP9:DTX3L expression and purification, pFastBac1 plasmids containing His_6_-DTX3L and MBP-PARP9 were transformed onto EmBacY cells to prepare bacmid for Sf9 cell infection. Suspension Sf9 cells were grown in Sf-900 II SFM (Gibco) at 27°C, 110 rpm, and were co-infected with His_6_-DTX3L and MBP-PARP9. Two days after proliferation arrest, the cells were harvested and resuspended in lysis buffer (20 mM HEPES pH 7.5, 200 mM NaCl, 0.5 mM TCEP, 5% glycerol, protease inhibitor cocktail (Roche), 0.5% CHAPS, 10 U/ml benzonase). The resuspended cells were lysed using a high-pressure homogeniser and centrifuged at 17 000 rpm at 4°C for 1 h. The clarified lysate was passed through pre-equilibrated HisTrap FF column (Cytiva). The column was washed in 20 mM HEPES pH 7.5, 200 mM NaCl, 20 mM imidazole, 0.5 mM TCEP, 5% glycerol. Bound proteins were eluted in 20 mM HEPES pH 7.5, 200 mM NaCl, 250 mM imidazole, 0.5 mM TCEP, 5% glycerol. The pooled elution fractions were then loaded onto pre-equilibrated amylose resin and the resin was washed in 20 mM HEPES pH 7.5, 200 mM NaCl, 0.5 mM TCEP, 5% glycerol. The His_6_-DTX3L:MBP-PARP9 complex was eluted in 20 mM HEPES pH 7.5, 200 mM NaCl, 10 mM maltose, 0.5 mM TCEP, 5% glycerol. The complex was further purified by size exclusion chromatography on a Superdex 200 column (GE Healthcare) in 20 mM HEPES pH 7.5, 200 mM NaCl, 0.5 mM TCEP, 5% glycerol. Peak fractions containing the complex were pooled and concentrated using a 30 kDa Viva Spin concentrator (Sartorius). Protein concentration was determined by *A*_280_ measurement using Nanodrop and calculated based on molar extinction coefficients. Proteins were aliquoted, snap frozen in liquid N2, and stored in −80°C freezer.

### Peptides

Histone 3 (H3) peptides used in this study were kind gifts from Matic Lab.

H3 peptide:

Ac-ARTKQTARKSTGGKAPRKQLAGGK(biotin)-Am

H3 S10-ADPr peptide:

Ac-ARTKQTARKS(ADPr)TGGKAPRKQLAGGK(biotin)-Am

### Oligonucleotide

Single-stranded RNA or DNA (ssRNA or ssDNA) oligos used in this study were commercially ordered from Invitrogen. Oligonucleotides were dissolved in 20 mM HEPES–KOH pH 7.6 and 50 mM KCl buffer.

Sequence of oligonucleotides used in this study (5′→3′):

E15_5′P_RNA [Phos]GUGGCGCGGAGACUU

E15_5′P_DNA [Phos]GTGGCGCGGAGACTT

E35_5′P_RNA_3Cy3 [Phos]GUGGCGCGGAGACUUAGAGAAAUUGUGAUCAGGAA[Cy3]

### NAD^+^ ubiquitylation assay

NAD^+^ ubiquitylation assays were performed as previously described. Briefly, (0.04–0.4 μM PARP9:DTX3L or 1 μM DTX2 RING-DTC or 1 μM DTX3L RING-DTC was incubated with 0.5 μM UBE1, 2.5 μM UBCH5A, 10 μM Ub and 50 μM NAD^+^ spiked with ^32^P NAD^+^ in 50 mM HEPES pH 7.5, 50 mM NaCl, 5 mM MgCl_2_, 1 mM DTT and 1 mM ATP. Reactions were stopped by addition of LDS sample buffer (Life Technologies) and incubation at 95°C for 5 min. Samples were then analyzed by an SDS-PAGE gel, which was stained with Coomassie staining and dried for autoradiography.

### Ub-NAD^+^ hydrolase assay

To obtain Ub-NAD^+^, 1 μM DTX3L RING-DTC was incubated with 0.5 μM UBE1, 2.5 μM UBCH5A, 10 μM Ub, and 50 μM NAD^+^ spiked with ^32^P-NAD^+^ in ubiquitylation buffer (50 mM HEPES pH 7.5, 50 mM NaCl, 5 mM MgCl_2_, 1 mM DTT and 1 mM ATP) at 37°C for 30 min, Then, indicated hydrolases (ADPr hydrolases, SARS-CoV-2 PLpro, and USP2) were added for a further 30 min treatment. All reactions were stopped by addition of SDS-PAGE loading dye and sample boiling. The samples were resolved by SDS-PAGE and analyzed by Coomassie staining and autoradiography.

### HPLC–MS analyses

HPLC–MS analyses were carried out on an Agilent 1260 Infinity HPLC system, coupled with an Agilent 6120 mass spectrometer [electrospray ionization (ESI) + mode]. The multiply charged envelope was deconvoluted using the charge deconvolution tool in Agilent OpenLab CDS ChemStation software to obtain the average [M] value.

### HPLC–MS monitoring of Ub-ADPr- by DTX3L RING-DTC or PARP9:DTX3L

Ub-ADPr was generated by incubation of 4 mM ADPr with 5 μM DTX3L RING-DTC or 1 μM PARP9:DTX3L (MBP-PARP9/His_6_-DTX3L), 0.5 μM UBE1, 2.5 μM UBCH5A and 24 μM Ub in 50 mM HEPES (pH 7.5), 50 mM NaCl, 5 mM MgCl_2_, 1 mM DTT and 2 mM ATP. Post incubation at 37°C for 30 min, 10 μl reactions were mixed with 2 μl of 1% TFA.

For NUDT16 treatment, prior to addition of 1% TFA, the reactions were treated with 4 μM NUDT16 and 10 mM MgCl_2_ (needed for the full activity of NUDT16) for 45 min at 37°C. Then reactions were subjected to HPLC–MS analysis as previously described (12).

### Electrophoretic mobility shift assay (EMSA)

The DTX3L RD or DTX3L full length proteins at varying concentrations were incubated in 10-μl reactions with 0.5 μM E35_5′P_RNA_3Cy3 in the buffer containing 20 mM HEPES (pH 7.5), 50 mM KCl, 5 mM MgCl_2_ and 1 mM DTT. After incubation on ice for 0.5 h, the reaction mixtures were separated on a pre-run 6% native PAGE gel and then the gel was imaged using the Molecular Imager PharosFX system (BioRad) with laser excitation for Cy3 at 532 nm.

### *In vitro* protein ADP-ribosylation assay

For PARP1 E988Q auto-modification, 0.5 μM PARP1 E988Q was incubated in the reaction buffer (50 mM Tris–HCl pH 8.0, 100 mM NaCl, 2 mM MgCl_2_, 1 μM DNA duplex (annealed from 5′-ATCAGATAGCATCTGTGCGGCCGCTTAGGG-3′ and 5′-CCCTAAGCGGCCGCACAGATGCTATCTGAT-3′), and 50 μM NAD^+^ spiked with ^32^P NAD^+^) at 37°C for 30 min and reactions were stopped by addition of 1 μM PARP inhibitor Olaparib.

### NUDT16 cleavage assay

Ub-NAD^+^ (generated by DTX2 RING-DTC or DTX3L RING-DTC or PARP9:DTX3L) was incubated with NUDT16 (complemented with 10 mM MgCl_2_) or NH_2_OH at 37°C for 1h. Auto-modified PARP1 E988Q was incubated with NUDT16 (complemented with 10 mM MgCl_2_) at 37°C for 1 h. The reactions were stopped by addition of the LDS sample buffer (Life Technologies) and incubation at 95°C for 5 min. Samples were then analyzed by SDS-PAGE and autoradiography.

### Ubiquitylation of ADP-ribosylated peptide

2 μg of H3 S10-ADPr peptide or a control unmodified H3 peptide was mixed with 1 μM DTX3L RING-DTC, 0.5 μM UBE1, 2.5 μM UBCH5A and 10 μM Ub in 50 mM HEPES pH 7.5, 50 mM NaCl, 5 mM MgCl_2_, 1 mM DTT and 1 mM ATP. After incubation at 37°C for 30 min, the reaction mixtures were resolved by SDS-PAGE, stained with Coomassie brilliant blue, and analyzed by western blotting. For ARH3 treatment, 2 μM ARH3 WT or ARH3 D77/78N were added to the reactions, and further incubated for 30 min at 37°C. Western blotting using anti-Ub (sc-8017, Insight Biotechnology) and anti-biotin (ab7403, Abcam) antibodies was performed to probe the ubiquitylated products.

### Ubiquitylation of ADP-ribosylated RNA and DNA

To generate ADP-ribosylated RNA or DNA, 50 μM RNA or DNA oligonucleotides were incubated with 20 μM PARP14 WWE-CAT (residues 1459–1801) or PARP10CAT (residues 868–1025) and 10 mM NAD^+^ in ADP-ribosylation buffer (20 mM HEPES–KOH pH 7.6, 5 mM MgCl_2_ and 1 mM DTT). The reactions were incubated at 37°C for 2 h and stopped by adding 50 ng/μl Proteinase K and 0.15% SDS followed by incubating at 50°C for 30 min. Then, the reaction mixture was incubated at 95°C for 5 min to inactivate Proteinase K. The reaction was further passed onto pre-equilibrated G25 column to get rid of the excess NAD^+^. The flow through was the mixture of modified and unmodified RNA or DNA and used for ubiquitylation reactions.

The flow through was incubated with 0.5 μM UBE1, 2.5 μM UBCH5A, 3 μM DTX3L RING-DTC and 10 μM Ub in 50 mM HEPES pH 7.5, 50 mM NaCl, 5 mM MgCl_2_, 1 mM DTT and 1 mM ATP. The reactions were incubated at 37°C for 1h and stopped by addition of 2× TBE urea sample buffer (8 M urea, 20 μM EDTA pH 8.0, 20 μM Tris–HCl pH 7.5, and bromophenol blue) and loaded on a pre-run 15% denaturing urea PAGE gel. The gels were run at 7 W/gel and following visualization under UV light (340 nm) after ethidium bromide (EB) staining.

## Results

### The RD tandem domain of DTX3L and full-length PARP9:DTX3L show the NAD^+^/ADPr ubiquitylation activity

Using DTX2 RD as a representative, we previously demonstrated that DELTEX E3 ligases can ubiquitylate NAD^+^/ADPr on the 3′ hydroxyl group of adenine-proximal ribose of NAD^+^/ADPr in the presence of Ub processing components (E1, E2, Ub and ATP) (12). We now again reproduced this result using NAD^+^ spiked with ^32^P NAD^+^, observing that DTX2 RD catalysed incorporation of radioactivity from NAD^+^ into Ub, as expected for NAD^+^ ubiquitylation (Figure 1D, lane 8).

To ask if the results obtained with DTX2 RD can be extended to the RD fragment of another DELTEX family member, DTX3L, we conducted a similar reaction but with DTX3L RD instead of DTX2 RD. Consistent with our previous study, DTX3L RD incorporated radioactivity from ^32^P NAD^+^ into Ub (Figure 1B, lane 1), suggesting that either the entire NAD^+^ or its part harbouring the labeled moiety (adenosine-proximal phosphate) became coupled to Ub (12). To clarify the chemical nature of the Ub adduct, the reactions were treated with two types of hydrolases: ADPr hydrolases and DUBs. All the ADPr hydrolases failed to remove the radioactivity from the Ub adduct, whilst two DUBs, SARS-CoV-2 PLpro and USP2, achieved complete removal (Figure 1B). The hydrolase sensitivity profile of the product of the DTX3L RD-catalysed reaction is thus identical to that observed for the DTX2 RD and consistent with NAD^+^ ubiquitylation but not with Ub ADP-ribosylation (12). Since DTX2 can use ADPr instead of NAD^+^ as a substrate, we tested if DTX3L can also accept ADPr. After incubating DTX3L RD with ADPr and Ub processing components, we tracked the product using high-performance liquid chromatography coupled to mass spectrometry (HPLC-MS). In line with the above speculation, we detected a mass that is consistent with the Ub-ADP-ribose adduct (9106 Da), strongly suggesting that DTX3L RD ubiquitylates ADP-ribose (Table 1) (Supplementary Figure S1).

**Table 1.** HPLC–MS identification of products of ubiquitylation reactions performed with indicated enzymes and substrates

Enzymes	Substrate	Detected mass of product (relative abundance)	Identified product, theoretical average mass
PARP9:DTX3L, E1, E2	ADP-ribose	9104.4 Da (> 90%)	Ub-ADP-ribose, 9106.0 Da
PARP9:DTX3L, E1, E2, followed by NUDT16	ADP-ribose	8892.3 Da (70%)	Ub-AMP, 8893.9 Da
		9104.3 Da (21%)	Ub-ADP-ribose, 9106.0 Da
DTX3L RING-DTC, E1, E2	ADP-ribose	9104.45 Da (> 90%)	Ub-ADP-ribose, 9106.0 Da
DTX3L RING-DTC, E1, E2, followed by NUDT16	ADP-ribose	8892.3 Da (60%)	Ub-AMP, 8893.9 Da
		9104.3 Da (34%)	Ub-ADP-ribose, 9106.0 Da

Dominant detected masses and corresponding products are provided. Product identification and quantification was performed as described in (12).

To know whether DTX3L RD ubiquitylates NAD^+^/ADPr on the adenine-proximal ribose similarly to DTX2 RD, we treated DTX3L RD-catalysed ^32^P-Ub-NAD^+^ with NUDT16. NUDT16 can cleave the pyrophosphate bond in the ADPr moiety, separating the adenine-proximal and adenine-distal ribose rings and their adjacent phosphates in such a way that the radioactive signal remains linked to the adenine-proximal part (58). The automodified PARP1 E988Q and DTX2 RD-catalysed ^32^P-Ub-NAD^+^ were treated with NUDT16 as controls. Additionally, as a control, the ^32^P-Ub-NAD^+^ created in a reaction catalyzed by DTX2 RD was treated with NH_2_OH to cleave the ester bond between Ub Gly^76^ and ^32^P NAD^+^, thus removing the radioactive signal. Consistent with previous data, NUDT16 treatment cleaved the radioactive signal off from auto-modified PARP1 E988Q but did not remove it from Ub attached to NAD^+^ using either DTX2 or DTX3L, while NH_2_OH reversed the radioactive signals completely (Figure 1D) (12). This is consistent with ADPr/NAD^+^ attachment to a protein via adenine-distal part in the case of PARP1 (canonical ADP-ribosylation) and through adenine-proximal part in the case of DTX-catalysed Ub modification (NAD^+^ ubiquitylation) (Figure 1C). We performed a similar experiment with unlabeled ADPr as a substrate, first generating Ub-ADP-ribose through a DTX3L RD-catalysed reaction, then cleaving it with NUDT16, and using HPLC-MS to characterize the product after NUDT16 treatment. The determined mass of the dominant species was consistent with Ub-adenosine monophosphate (AMP), as expected for the initial Ub attachment being to the ADPr's adenine-proximal ribose (Table 1) (Supplementary Figure S2).

It has not been tested before if the ADPr/NAD^+^ ubiquitylation activity observed for DTX2 RD and DTX3L RD can also be detected using a full-length version of any DELTEX family member, where domains other than the RD could potentially affect the course of the reaction. In particular, the formation of a tight complex between DTX3L and its binding partner, PARP9 (a potential ADP-ribosyltransferase), introduces additional domains from both proteins that could potentially affect the activity. Indeed, it has been a matter of debate whether PARP9:DTX3L catalyses Ub ADP-ribosylation (45,50,53,54) or ubiquitylation of NAD^+^ (Figure 1A) (12). To resolve this question, we first reproduced previous results (45,50,53), demonstrating that PARP9:DTX3L incorporated radioactivity from ^32^P NAD^+^ into Ub in the presence of Ub processing components (E1, E2 and ATP) (Supplementary Figure S5A). Worth noting, PARP9:DTX3L did not show auto-ADP-ribosylation in the presence of ^32^P NAD^+^, which, considering that active PARPs generally auto-ADP-ribosylate, might imply that the complex is not active in ADP-ribosylation (Figure 1D, lane 5). Next, we subjected the reactions to hydrolysis by ADPr hydrolases and one DUB. Consistent with the result obtained with DTX3L RD, all the tested ADPr hydrolases failed to remove the radioactive signal from the Ub adduct, whilst one DUB, SARS-CoV-2 PLpro, completely removed it (Supplementary Figure S5B). To further confirm that the PARP9:DTX3L complex exhibits the same NAD^+^/ADPr ubiquitylation activity as the RD fragments of DTX2 or DTX3L, we incubated unlabelled ADPr with PARP9:DTX3L, E1, E2 and ATP, and subjected it to MS analysis. As expected, the dominant detected mass was in good agreement with the calculated mass of Ub-ADPr. Furthermore, NUDT16-mediated processing of this product resulted in a detected mass corresponding to Ub-adenosine monophosphate (AMP), which is in line with Ub attached to the adenine-proximal ribose of the ADP-ribose (Table 1) (Supplementary Figures S3 and S4).

Collectively, these findings strongly suggest that PARP9:DTX3L catalyses ubiquitylation of NAD^+^/ADPr on the adenine-proximal ribose ring, presumably via the 3′ hydroxyl group of that ring, as previously characterised for the DTX2 RD-catalysed reaction. A similar result obtained with the full-length PARP9:DTX3L complex and RD fragments of DTX3L and DTX2 suggests that the presence of additional domains in DTX3L and PARP9 does not impinge on the ability of the RD tandem domain—a region shared by all DELTEX proteins—to ubiquitylate the ADPr moiety. However, it is probable that these additional domains affect the selection of substrates and the execution of this activity within the cell. This applies in particular to the macrodomain 1 of PARP9, which has recently been shown to have an ADPr hydrolase activity (43,59,60).

In the rest of the present study, we focused on the isolated DTX3L RD fragment in order to elucidate the inherent catalytic potential that could be modulated by additional domains in the full-length complex.

### DTX3L RD ubiquitylates ADP-ribosylated peptides

**Figure 2.**
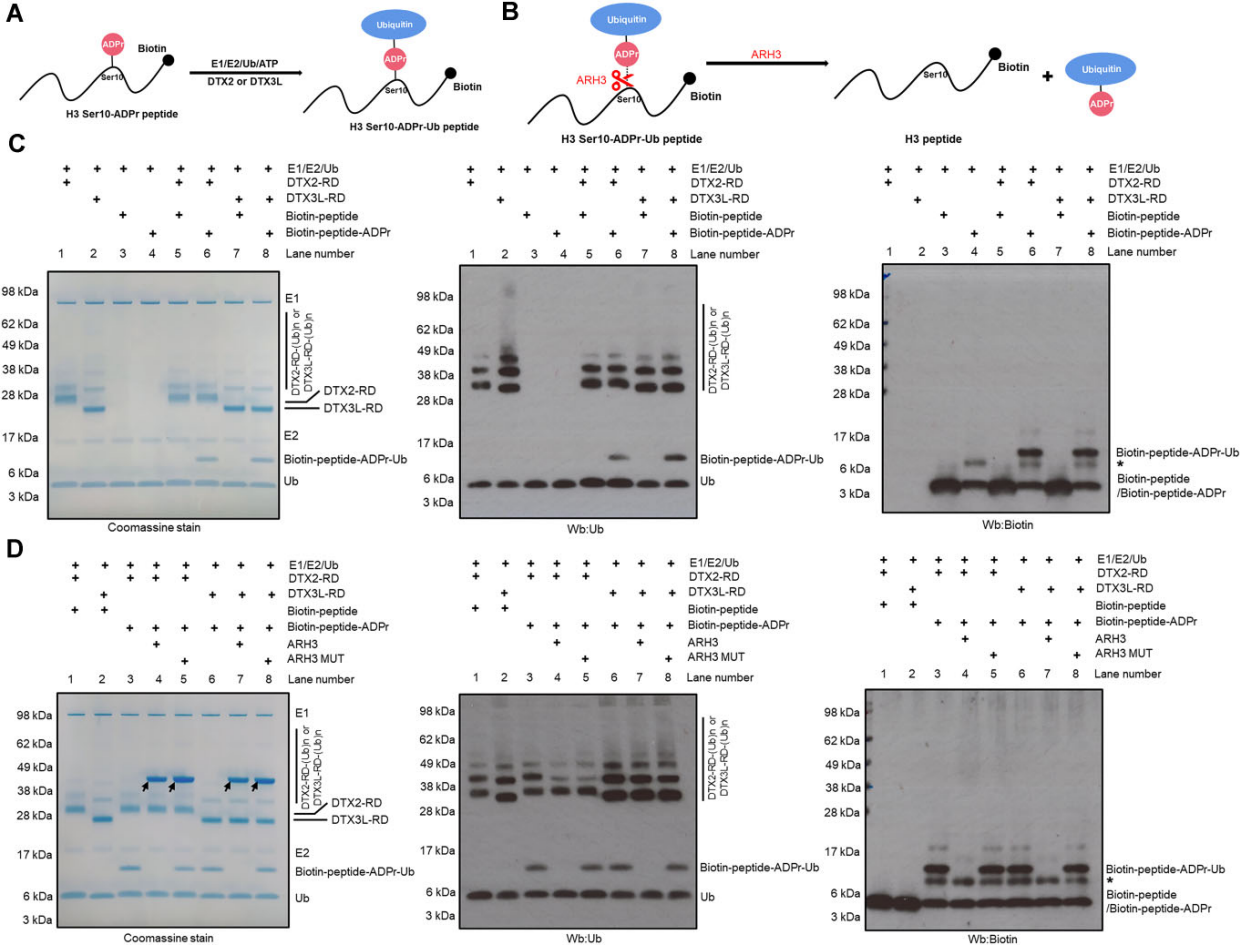
DTX3L-RD ubiquitylates ADP-ribosylated peptides on the ADPr moiety. (**A**) DTX3L-RD ubiquitylated H3 S10-ADPr peptide on the ADPr moiety, forming a combined Ub-ADPr- modification. (**B**) A schematic showing the cleavage site of ARH3 within H3 S10-Ub-ADPr- peptide. (**C**) The H3 S10-Ub-ADPr- product was obtained by incubation of H3 S10-ADPr peptide with either DTX2 RD or DTX3L RD in presence of Ub, E1, E2 and ATP, as illustrated in (A). The same reaction with an unmodified H3 peptide was performed as a control. The samples were analysed by SDS-PAGE, next visualized by Coomassie staining (left) and western blotting (Wb) with anti-Ub (middle) or anti-biotin (right; detecting biotinylated peptides) antibodies. The asterisk represents a contaminant present in H3 S10-ADPr peptide. (**D**) As in (C), the H3 S10-Ub-ADPr- products were treated with either ARH3 WT or ARH3 MUT, revealing sensitivity of the H3 S10-Ub-ADPr- adduct to ARH3, consistent with ubiquitylation of H3 S10-ADPr on the ADPr moiety as illustrated in (B). The samples were analyzed by SDS-PAGE, next visualized by Coomassie staining (left) and western blotting (Wb) with anti-Ub (middle) or anti-biotin (right; detecting biotinylated peptides) antibodies. The arrows indicate WT and mutant ARH3, while the asterisk represents a contaminant present in H3 S10-ADPr peptide.

In our previous study, we demonstrated that DTX2 RD can ubiquitylate not only free NAD^+^ or ADPr but also an ADPr moiety that is covalently attached to a peptide or a protein, as in the case of protein ADP-ribosylation catalysed by PARPs. In light of this, we sought to investigate whether DTX3L RD is also capable of ubiquitylating ADPr on a peptide. To test this, we used, as a substrate, a biotinylated version of either a histone H3-derived peptide that is modified on serine 10 with a single ADPr moiety (H3 S10-ADPr peptide, prepared as described in (28,61)) or the same peptide without ADPr (H3 peptide). In the presence of Ub processing components, DTX3L RD and DTX2 RD ubiquitylated H3 S10-ADPr peptide (shown as an upward shift of a part of the Ub on SDS-PAGE), but not the control peptide without ADPr modification (Figure 2A, C). Western blotting using anti-biotin (to label the peptide) and anti-Ub antibodies confirmed that the shifted bands correspond to a ubiquitylated H3 S10-ADPr peptide. To distinguish between two scenarios: (i) ubiquitylation on one of the lysine residues within the H3 S10-ADPr peptide or (ii) ubiquitylation on the ADPr moiety attached to a serine within the H3 S10-ADPr peptide, we tested the sensitivity of the product to hydrolysis by ARH3, which specifically reverses serine ADP-ribosylation (Figure 2B) (25). ARH3 but not its catalytic mutant caused the disappearance of the higher band, confirming that Ub was attached to the H3 S10-ADPr peptide via the ADPr moiety, and likely through its adenine-proximal 3′ ribose hydroxyl group, as shown for DTX2 RD (Figure 2D). Taken together, our data showed that DTX3L RD and likely the full-length PARP9:DTX3L can ubiquitylate ADP-ribosylated peptides and proteins.

### DTX3L contains multiple putative nucleic acids binding domains

**Figure 3.**
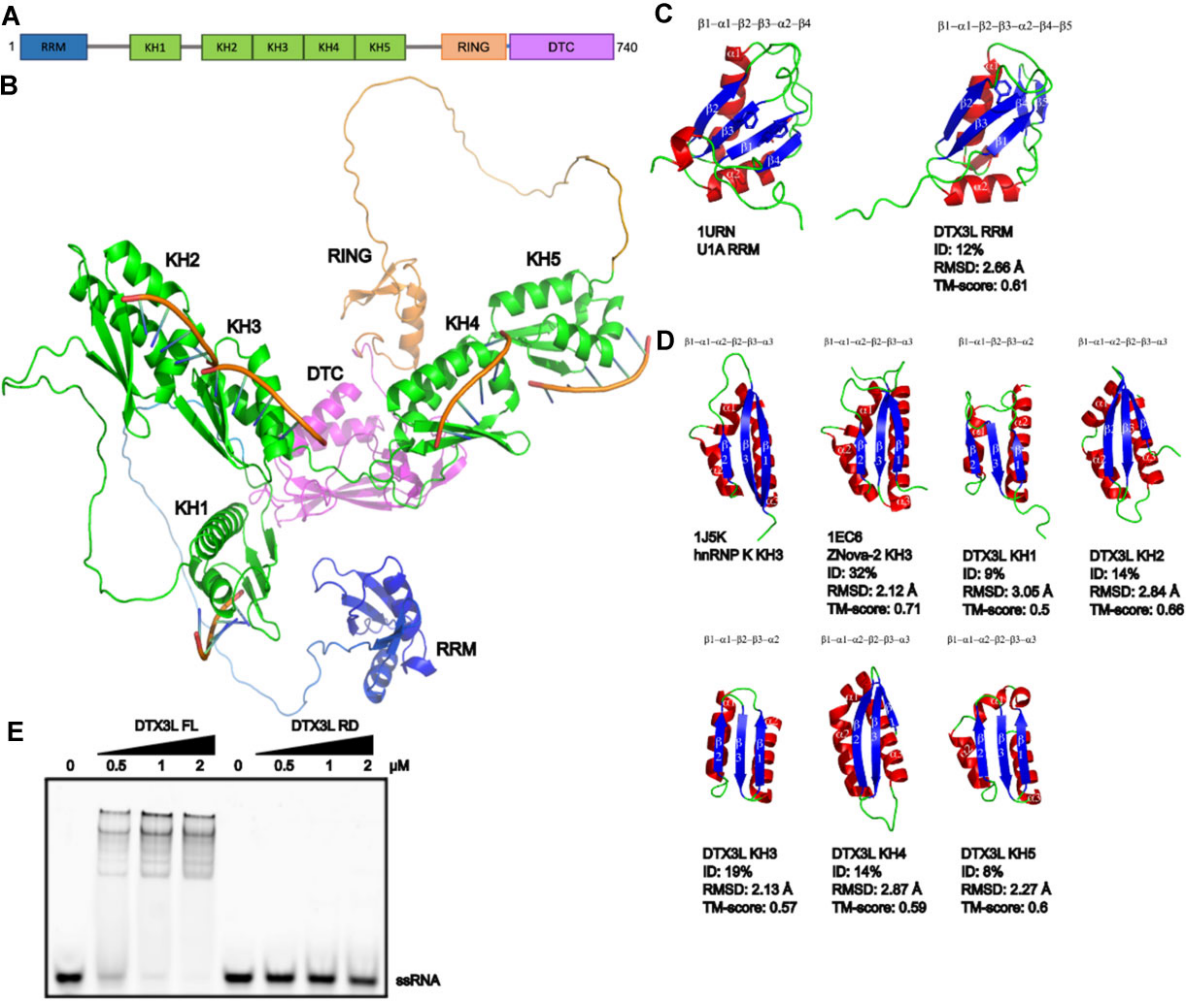
Structural analysis shows that DTX3L harbors putative single-stranded nucleic acids binding domains. (**A**) The schematic diagram showing the domain organization of DTX3L. (**B**) Cartoon representation of DTX3L structure as predicted by AlphaFold (62,63). The domains are colored as follows: RRM in blue, KH in green, RING in orange and DTC in magenta. ssRNA is modelled by aligning the model with hnRNP K KH3 domain with bound ssRNA (PDB: 1J5K). (**C**) RRM domain from DTX3L compared to RRM domain from U1A using pairwise structure alignment. Helices are colored in red, sheets in blue and loop in green. (**D**) Individual KH domains from DTX3L and Nova-2 compared to KH3 from hnRNP K using pairwise structure alignment. Predicted KH domains of DTX3L have low sequence identity making it difficult to identify using sequence alignment. However, the structures align reasonably well with root mean square deviation (RMSD) values around 2–3 Å. Template modeling score (TM-score) of ∼0.5–0.6 indicates that these domains generally have the same fold. Colored as in (C). (**E**) Electrophoretic mobility shift assay (EMSA) of one ssRNA oligonucleotide with DTX3L full length protein or DTX3L RD protein (increasing concentrations of 0.5, 1 and 2 μM).

Benefiting from the recently developed powerful tool AlphaFold2, which predicts protein structure with a high accuracy (62,63), we analyzed the structural model of DTX3L and discovered previously unannotated structural domains. In addition, we recently analysed in a similar manner PARPs including PARP9 (43). According to high-confidence AlpahFold2 predictions, DTX3L has multiple nucleic acids binding domains, none of which has been previously reported: one RNA recognition motif (RRM) domain and five K homology (KH) domains preceding the RING and DTC domains (Figure 3A, B).

The DTX3L RRM domain contains two alpha helices and four antiparallel beta strands organized in β_1-_α_1-_β_2-_β_3-_α_2-_β_4_ topology, which superimposes well with RNA-binding domain of the small nuclear ribonucleoprotein U1A in complex with a 21-nucleotide RNA hairpin, with a Root Mean Square Deviation (RMSD) of ∼2.66 Å (Figure 3C). A canonical RRM domain has conserved aromatic residues lining the β_1_ and β_3_ strands (RNP2 and RNP1 motifs), through which they form stacking interactions with RNA bases to achieve sequence specificity (64). In DTX3L’s RRM, there is only one aromatic residue on the β_3_ strand, and hydrophobic non-aromatic residues on the β_1_ strand (Figure 3C), which may indicate a noncanonical way of binding nucleic acids or involvement in protein:protein interactions. Further investigation is needed to address this question.

In addition to the RRM domain, DTX3L possesses five KH domains, which align reasonably well with human heterogeneous nuclear ribonucleoprotein (hnRNP K) (65) with RMSD values around 2–3 Å (Figure 3D). The KH domain was first identified in the hnRNP K and usually binds four-nucleotide-long stretches of ssRNA or ssDNA in a sequence-specific manner (66). There are two types of KH domain folds with similar structure but different topology: type I and type II (67). Type I KH domain is typically found in eukaryotic proteins, while type II KH domain is widely found in prokaryotic proteins. Consistent with this, all five KH domains in human DTX3L are type I and adopt β_1_-α_1_-α_2_-β_2_-β_3_-α_3_ topology, except for KH1 and KH3 that are one helix short (Figure 3D). The RRM domain and the first KH domain in DTX3L are predicted to be connected via a long linker, while the other four consecutive KH domains are more rigid, with tandem KH2-KH3 and tandem KH4-KH5 possibly accommodating a stretch of >16 nucleotide long ssRNA or ssDNA (Figure 3B).

To test whether DTX3L has RNA binding ability, we performed an electrophoretic mobility shift assay (EMSA) using DTX3L RD and full-length DTX3L and an ssRNA oligonucleotide. As shown in Figure 3E, DTX3L RD did not display any noticable binding to ssRNA. In contrast, full length DTX3L bound to ssRNA, indicating that the RRM domain and/or KH domains present within the full-length construct possess RNA binding property.

The presence of multiple potential nucleic acid-binding domains in DTX3L indicates a close link between this protein and nucleic acid-associated cellular processes. Noteworthy, the N terminal part of DTX3L resembles that of PARP9 and PARP14 (Supplementary Figure S6), of which the latter harbors several RRM and KH domains and was recently shown to ADP-ribosylate phosphorylated nucleic acids (43).

### DELTEX E3s ubiquitylate ADP-ribosylated nucleic acids

**Figure 4.**
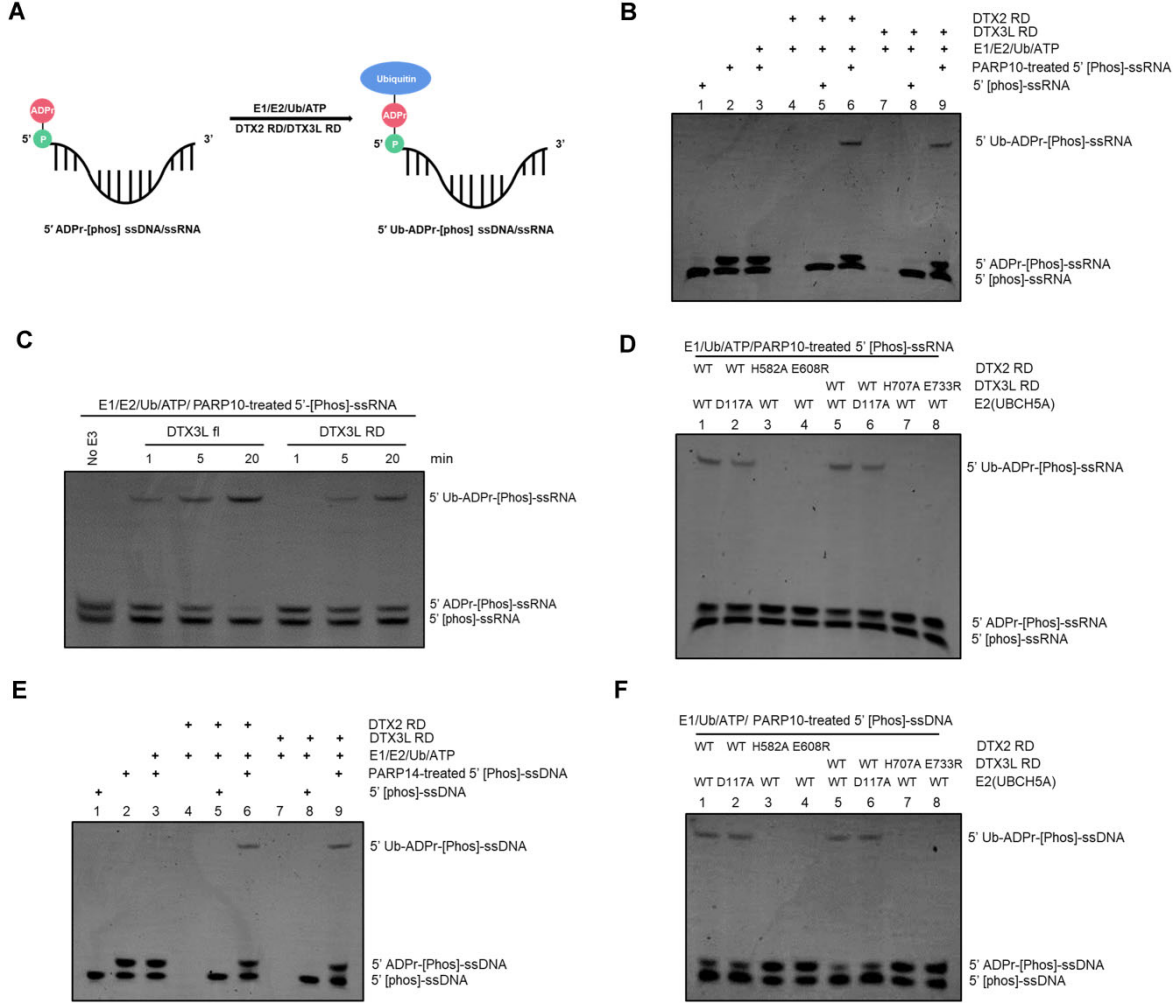
DTX3L-RD ubiquitylates ADP-ribosylated nucleic acids on the ADPr moiety. (**A**) A schematic showing the ubiquitylation of ADP-ribosylated nucleic acids. DTX2 RD/DTX3L-RD ubiquitylated 5′ ADP-ribosylated nucleic acids on the ADPr moiety, forming a combined Ub-ADPr- modification. (**B**) Biochemical reconstitution of the ubiquitylation of ADP-ribosylated ssRNA. ADP-ribosylated and (or) unmodified ssRNA was incubated with either DTX2 RD or DTX3L RD in the presence of Ub processing components (E1, E2, Ub and ATP). The samples were analyzed on a urea gel, which is then visualized under UV light (340 nm) after ethidium bromide (EB) staining. (**C**) DTX3L full length protein ubiquitylated ADP-ribosylated ssRNA with stronger efficacy than DTX3L RD does. ADP-ribosylated and (or) unmodified ssRNA was incubated with either DTX3L fl or DTX3L RD in the presence of Ub processing components (E1, E2, Ub and ATP). The reactions were stopped at indicated times and analyzed on a urea gel, which is then visualized under UV light (340 nm) after ethidium bromide (EB) staining. (**D**) DTX2 RD/DTX3L RD ubiquitylated 5′ ADP-ribosylated ssRNA on the ADPr moieties. Asp^117^ of UbCH5A (E2), which is defective of catalyzing amine group ubiquitylation (68,69), is used to show that DTX2 RD and DTX3L RD ubiquitylated the hydroxyl groups in ADP-ribosylated nucleic acids. DTX2 and DTX3L ADPr ubiquitylation inactive mutants failed to produce upshift bands that correspond to ubiquitylation of ADP-ribosylated nucleic acids, confirming that Ub is attached to ADPr moiety. The samples were analyzed on a urea gel, which is then visualized under UV light (340 nm) after ethidium bromide (EB) staining. (**E**) As in (B), ssDNA was used as substrate for ubiquitylation assay. (**F**) As in (D), ubiquitylated ADP-ribosylated ssDNA was used as substrate for hydrolysis assay.

Considering that DTX3L has domains that could allow it to bind nucleic acids, we wondered whether the catalytic RD tandem domain of DTX3L can ubiquitylate ADP-ribosylated nucleic acids in addition to free NAD^+^/ADPr and ADP-ribosylated peptides or proteins (Figure 4A). If such an activity was present in the RD of DTX3L, it could conceivably be targeted to specific nucleic acid substrates by the binding domains discussed above.

Recently, several studies revealed that certain PARPs, including PARP10 and PARP14, can ADP-ribosylate phosphorylated nucleic acids (33,39–43). Therefore, we took advantage of PARP10 and PARP14 to generate ADP-ribosylated nucleic acids by incubating catalytic fragments of these PARPs with 5′ phosphorylated forms of ssRNA or ssDNA, respectively. In both cases, we obtained an approximately 1:1 mixture of modified (ADP-ribosylated) and unmodified ssRNA or ssDNA, which we then used as a substrate for ubiquitylation. To discriminate between the potential ubiquitylation of unmodified and modified ssRNA or ssDNA, we introduced control reactions in which only unmodified nucleic acids were present. To extend the investigation to other DELTEX family members beyond DTX3L, we tested DTX2 RD in parallel to DTX3L RD.

Treating mixtures of ADP-ribosylated and unmodified ssRNA obtained from PARP reactions with DTX3L RD or DTX2 RD in the presence of E1, E2 and ATP led to the appearance of upward-shifted bands (Figure 4B, lane 6 and lane 9), indicating that either ADP-ribosylated or unmodified ssRNA became ubiquitylated. However, the reactions with only unmodified ssRNA did not show any higher bands, speaking against the ability of unmodified ssRNA to be ubiquitylated by DTX RD fragments (Figure 4B, lane 5 and lane 8). Thus, DELTEX-mediated ssRNA ubiquitylation appears to be ADPr-dependent and the upper band observed upon a mixture of modified and unmodified ssRNA can be attributed to ubiquitylation of ADP-ribosylated ssRNA. Considering that DTX3L contains multiple nucleic acids binding domains and, as shown above, binds to ssRNA, we asked whether full-length DTX3L ubiquitylates ADP-ribosylated ssRNA more efficiently than DTX3L RD does. Consistent with our expectations, full-length DTX3L was markedly more efficient than its RD fragment. This is likely due to the ssRNA binding ability, which helps DTX3L recruit an ADP-ribosylated ssRNA substrate (Figure 4C).

We previously showed that DTX2 RD-mediated NAD^+^/ADPr ubiquitylation–unlike conventional ubiquitylation on protein lysine residues, is not strongly dependent on the catalytic D117 residue in the E2 enzyme, UBCH5A, used in the reaction (12,68,69). We attributed this to the fact that the DTC domain of DELTEX proteins contains catalytic histidine and glutamate residues (H582 and E608 in DTX2, H707 and E733 in DTX3L) (Supplementary Figure S7) that can work together to deprotonate and thus activate the acceptor 3′ hydroxyl group in the ADPr moiety–a task that in the case of a canonical lysine acceptor is likely assisted by E2’s D117 residue. A multiple sequence alignment of the DTC domains from the DELTEX family members revealed that the catalytic histidine and glutamate residues are strictly conserved (Supplementary Figure S8), highlighting the conserved ADPr ubiquitylation property of DELTEX family E3 ligases. To shed light on the chemical nature of DELTEX-mediated ubiquitylation of ADP-ribosylated ssRNA, we tested if the reaction depends on D117 of E2. We observed that the ubiquitylation reaction catalysed by either DTX3L RD or DTX2 RD was efficient to a similar degree with WT and D117A UBCH5A (Figure 4D, lanes 1–2 and lanes 5–6). This hints at the ubiquitylation likely taking place on the 3′ hydroxyl group of the ADPr moiety that can be specifically activated by the DTC domain rather than on other nucleophilic groups within ssRNA, which would likely require the D117 residue. Consistent with this explanation, ubiquitylation of ADP-ribosylation ssRNA was abolished upon mutating catalytic histidine and glutamate residues present in the DTC domain of either DTX3L RD or DTX2 RD, which are required for ADPr ubiquitylation but not canonical lysine ubiquitylation (Figure 4D, lanes 1, 3 and 4, lanes 5, 7 and 8). Overall, these results strongly suggest that the observed ubiquitylation of ADP-ribosylated ssRNA happens on the ADPr moiety, likely through the 3′ hydroxyl group of adenine-proximal ribose.

In an analogous set of experiments, we reproduced the same observations with ADP-ribosylated ssDNA as a ubiquitylation substrate (Figure 4E and F). Briefly, both DTX3L RD and DTX2 RD could ubiquitylate modified but not unmodified ssDNA. The reaction was largely independent of D117 of E2 but required catalytic histidine and glutamate residues within DTX3L or DTX2 fragments.

Collectively, our data showed that DTX3L RD and DTX2 RD can ubiquitylate ADP-ribosylated nucleic acids on the ADPr moiety. This is the first demonstration of enzymatic ubiquitylation of nucleic acids (in this case, indirectly via ADPr).

### Distinct hydrolases reverse DELTEX E3s-catalysed ubiquitylation on the ADPr moiety of nucleic acids

**Figure 5.**
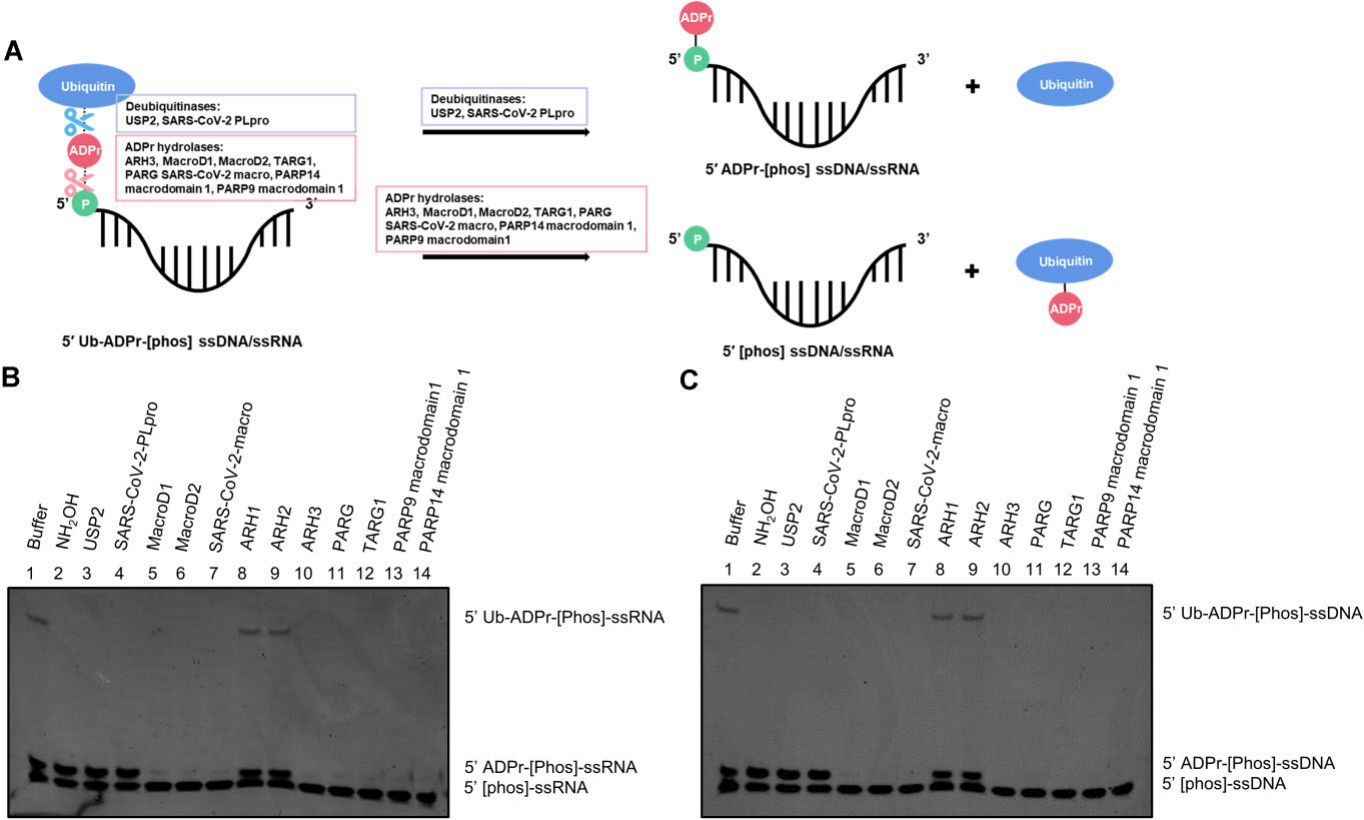
Reversal of ubiquitylation on ADP-ribosylated nucleic acids. (**A**) Schematic illustration showing that the Ub-ADPr- dual modification on phosphorylated nucleic acids can be removed by two distinct classes of hydrolases: ADPr hydrolases and DUBs. Due to different recognition sites within the ubiquitylated nucleic acids, treatment of these two types of hydrolases result in different outcomes. DUBs cleavage generate ADP-ribosylated nucleic acids and free Ub, while ADPr hydrolases remove the Ub-ADPr- and generate phosphorylated nucleic acids. (**B**) Hydrolysis of ubiquitylated ssRNA. Following the ubiquitylation of ADP-ribosylated ssRNA with DTX3L RD, the indicated ADPr hydrolases and DUBs were added and further incubated. The samples were analyzed on a urea gel, which is then visualized under UV light (340 nm) after ethidium bromide (EB) staining. (**C**) As in (B), ubiquitylated ssDNA was used as substrates for hydrolysis.

We next wanted to investigate the enzymatic reversal of the combined Ub-ADPr- modification on nucleic acids. The modification is achieved by the crosstalk between ADP-ribosylation and ubiquitylation, therefore, theoretically, it could be reversed by both ADPr hydrolases and DUBs, which would be expected to cleave off either the whole adduct or just the Ub unit, respectively (Figure 5A). We first used a catalytic PARP domain followed by DTX3L RD to generate a ubiquitylated form of ADP-ribosylated RNA (5′ Ub-ADPr-[Phos]-ssRNA). This product was treated with various ADPr hydrolases and DUBs. Among all the tested ADPr hydrolases, only ARH1 and ARH2 did not remove the Ub-ADPr-, while the other ones, including two newly identified ADPr hydrolases (PARP9 macrodomain 1 and PARP14 macrodomain 1) (59,60,70), completely reversed Ub-ADPr- from RNA substrates. In contrast—but as expected—both USP2 and SARS-CoV-2 PLpro only cleaved Ub off, leaving the ADPr moiety still attached to RNA (Figure 5B). Like before, the experiment was repeated with ssDNA instead of ssRNA, which led to the same conclusions (Figure 5C).

Taken together, these data demonstrate that the DELTEX-catalysed compound Ub-ADPr- modification on (phosphorylated) nucleic acids is reversible, with two groups of hydrolases, ADPr hydrolases and DUBs, being able to efficiently process it. Additionally, these experiments confirmed that DELTEX E3s attach Ub to an ADPr moiety on ADP-ribosylated nucleic acids.

## Discussion

DELTEX family E3s share a characteristic RING-DTC (RD) tandem domain at their C termini. We recently showed that, in the case of DTX2, this region can catalyse efficient Ub attachment onto the 3′ hydroxyl of adenine-proximal ribose in ADPr and ADP-ribosylated peptides and proteins *in vitro* (12). However, it has been unclear whether this mechanism is also employed by other E3 ligases from the DELTEX family and how their NAD^+^/ADPr ubiquitylation activities might be regulated in the full-length protein context in which other domains exist. This ambiguity has applied, in particular, to DTX3L, which not only, like other full-length DELTEX proteins, has multiple domains in addition to the RD region but also is unique in this family in forming a tight complex with a potential ADP-ribosyl transferase, PARP9. It was this complex that was initially demonstrated to catalyse a reaction between NAD^+^ and Ub (45). While PARP9 itself has been reported to be inactive, it has theoretically remained possible that it becomes activated in the presence of DTX3L, possibly through a provision of a missing catalytic residue, as in the case of PARP1 or PARP2 activation by HPF1 (71). In such a scenario, PARP9 could conceivably catalyse Ub ADP-ribosylation, while the RD fragment of DTX3L would not serve a catalytic role. To address this uncertainty, we now used biochemical and MS approaches to demonstrate that DTX3L RD and the full-length PARP9:DTX3L complex possess the same core NAD^+^/ADPr ubiquitylation activity that we described for the RD fragment of DTX2. Our data are not consistent with the ADP-ribosylation of Ub on its C terminus, which has been suggested as an alternative scenario (45,50,53).

In the cell, the ADPr moiety that DELTEX ligases can ubiquitylate is found not only within NAD^+^ and possibly free ADPr, but also in ADP-ribosylated substrates that became modified by PARP enzymes. For a long time, proteins have been considered sole substrates of enzymatic ADP-ribosylation, but recently more and more studies show that nucleic acids—in particular those with phosphorylated termini—can also become ADP-ribosylated (39–42). Given that we previously showed that DELTEX E3s can ubiquitylate the ADPr moiety in ADP-ribosylated proteins, we now asked if they are also able to ubiquitylate an ADPr moiety attached to nucleic acids. This question was further motivated by the fact that our current analysis of AlphaFold2 models suggested the presence of putative nucleic acids binding domains within DTX3L, whose ssRNA binding ability was confirmed using an EMSA assay (Figure 3A and E), suggesting that some DELTEX family members are involved in nucleic acids biology. Our experiments show that 5′-phosphorylated ssRNA or ssDNA molecules that have been pre-ADP-ribosylated by PARPs can be subsequently efficiently ubiquitylated by DTX3L RD or DTX2 RD *in vitro*. Moreover, the full-length DTX3L protein, which features multiple putative nucleic acids binding domains, was more efficient at catalysing ubiquitylation of ADP-ribosylated ssRNA than DTX3L RD (Figure 4C), likely due to the improved recruitment of the ssRNA substrate via these domains. Thus, our study demonstrates, for the first time, the ubiquitylation of nucleic acids, which in this case happens indirectly on an ADPr moiety, generating a combined Ub-ADPr- modification on DNA or RNA. It is worth noting that various types of ADP-ribosylated RNA exist in cells (33), which could potentially serve as substrates of DELTEX-mediated ubiquitylation. The function of the resultant Ub-ADPr- modification on nucleic acids might be as a signal for recruiting specific binding proteins to regulate downstream pathways or affect the stability of RNA or promote RNA degradation pathways.

Evolutionary studies see extensive crosstalk between ADP-ribosylation and ubiquitylation pathways (72), but these connections remain largely unstudied. The only well-characterized example is the interplay between PARP family members Tankyrase1/2 and the WWE domain-containing E3 ligases including RNF146. In the Wnt signaling pathway, Tankyrase1/2 modify Axin with PAR chains, which later recruit and allosterically activate RING E3 ligase RNF146 via binding to the WWE domain, resulting in the ubiquitylation of Axin on lysine residues and its subsequent degradation (73–76). Another example comes from PARP1 and Thyroid hormone Receptor Interacting Protein 12 (TRIP12), whereby the PAR chains on trapped PARP1 recruit TRIP12, which then ubiquitylates trapped PARP1, targeting it for degradation (77). In addition, we previously showed that the Ub-ADPr- dual modification can be efficiently created on proteins *in vitro* through a crosstalk between PARPs and DELTEX E3s (12). Here, we show that this modification can also be produced on nucleic acids. In addition to reconstituting this process *in vitro*, we identified hydrolases that are able to reverse it. Like previously in the case of Ub-ADPr- on protein substrates, we determined that when this dual modification is linked to nucleic acids, it can be cleaved both by several ADPr hydrolases (ARH3, PARG, MacroD1/2, TARG1, SARS-CoV-2 macro, as well as two recently identified macro domains: PARP9 macrodomain 1 and PARP14 macrodomain 1) and at least two DUBs (USP2 and SARS-CoV-2 PLpro). These two types of enzymes remove either the whole compound adduct or just the Ub part; in cells, they could be used in different contexts. Interestingly, PARP9 has recently been shown to possess, within its macrodomain 1, an ADPr hydrolase activity, which we now showed to be active against the Ub-ADPr- modification (59,60,70). This paradoxical coupling of Ub-ADPr- synthesis and removal functions within the PARP9:DTX3L complex could serve to fine-tune and proofread the produced modification.

Interestingly, our findings resonate with recent insights into the cellular response to viruses, particularly SARS-CoV-2. DTX3L, PARP10 and PARP14 are stimulated upon SARS-CoV-2 infection (78) and potentially carry out anti-viral roles, which could be explained by their sequential contribution to the synthesis of Ub-ADPr- on viral proteins or nucleic acids, possibly leading to virulence inhibition. Supporting this hypothesis, as shown in this study that DELTEXes work in conjunction with the main anticoronaviral PARP PARP14 to produce Ub-ADPr (79). Furthermore, SARS-CoV-2 and other coronaviruses encode nonstructural protein 3 (Nsp3), which contains both a DUB and an ADPr hydrolase activity and reside in the adjacent PLpro (80,81) and macro (82) domains, respectively (12). Since we showed that these two domains are individually active in processing Ub-ADPr-, the entire Nsp3 protein could efficiently reverse the Ub-ADPr- modification installed by PARP14 (or possibly other antiviral PARPs containing KH domains such as PARP10 (43,83,84)) and DTX3L, thus antagonizing their potential anti-viral function.

In summary, our data show that PARP9:DTX3L ubiquitylates ADPr on the adenine-proximal ribose moiety (presumably the 3′ hydroxyl), by the action of the RD tandem domain found in all DELTEX family E3s. Strikingly, we also demonstrate that DELTEX ligases can ubiquitylate ADP-ribosylated nucleic acids including ssRNA and ssDNA, generating the Ub-ADPr- dual modification on nucleic acids. This novel nucleic acid modification can be reversed by several ADPr hydrolases and DUBs. Future studies should determine the biological function of the uncovered reactions.

## Supplementary Material

gkad1119_Supplemental_File

## Data Availability

The data underlying this article are available in the article and in its online supplementary material.
