# Supplementary material for: DELTEX E3 ligases ubiquitylate ADP-ribosyl modification on nucleic acids
Source: Nucleic Acids Res. 2023 Nov 24;52(2):801–15. doi: 10.1093/nar/gkad1119 (PMC10810221; doi:10.1093/nar/gkad1119)
Supplement: gkad1119_Supplemental_File [file gkad1119_supplemental_file.pdf]

Supplementary Information

DELTEX E3 ligases ubiquitylate ADP-ribosyl modification on nucleic acids

Kang Zhu[1*], Marcin J Suskiewicz[2], Chatrin Chatrin[1], Øyvind Strømland[1, 3], Bryan W Dorsey[4], Vincent Aucagne[2], Dragana Ahel[1*], Ivan Ahel[1*]

[1]Sir William Dunn School of Pathology, University of Oxford, Oxford, UK.

[2]Centre de Biophysique Moléculaire, CNRS UPR 4301, Orléans, France.

[3]Department of Biomedicine, University of Bergen, Bergen, Norway.

[4]Ribon Therapeutics, 35 Cambridgepark Dr., Suite 300, Cambridge MA 02140, USA.

* Correspondence: kang.zhu@path.ox.ac.uk, dragana.ahel@path.ox.ac.uk, ivan.ahel@path.ox.ac.uk

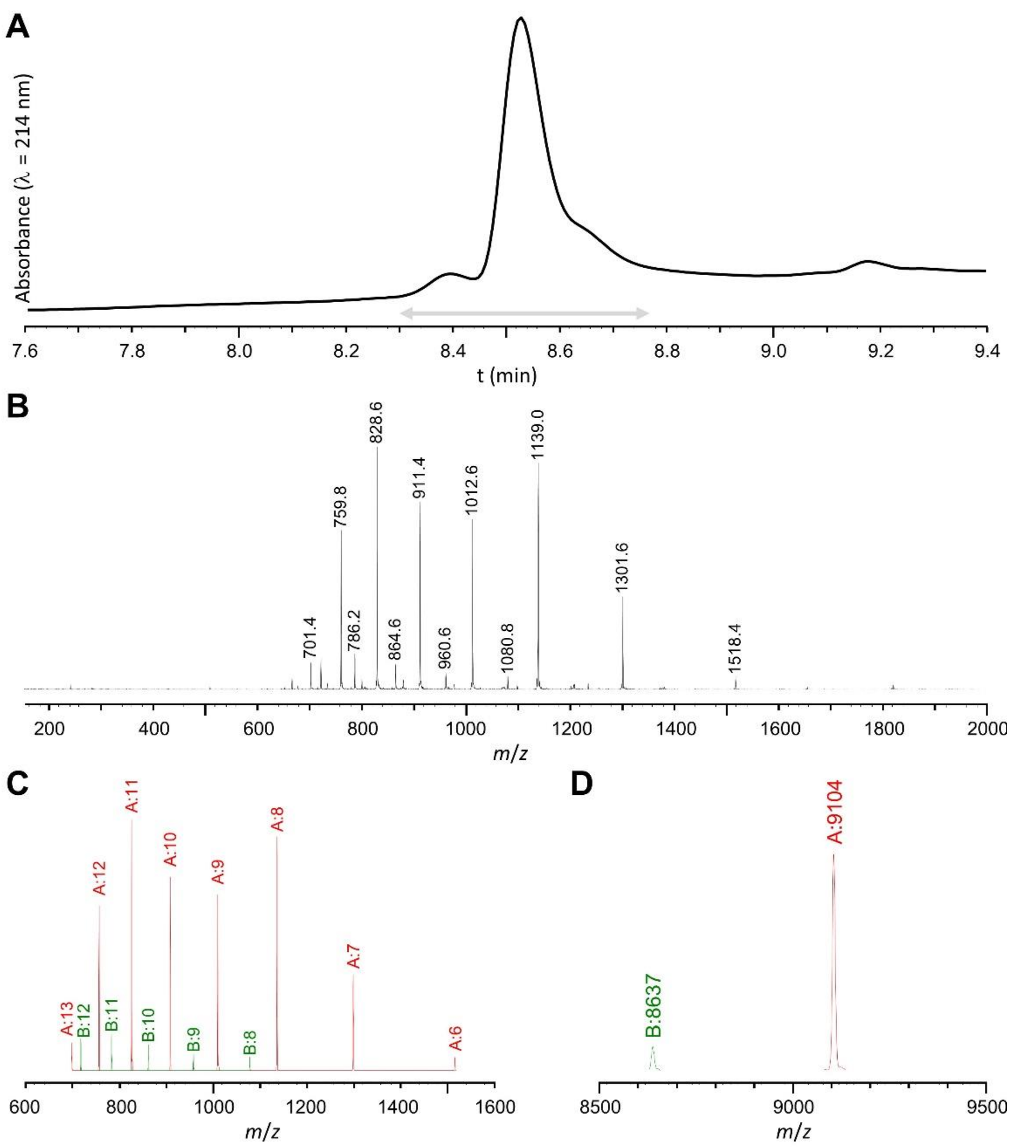

E

Compound	Relative abundance (%)	Detected average mass	Identified product, its theoretical average mass
A	90.1	9104.5 Da	Ub-ADP-ribose, 9106.0 Da
B	9.9	8637.0 Da	Ub-glycerol, 8638.8 Da

Figure S1 LCMS analysis of ADPr ubiquitylation mediated by DTX3L RING-DTC. **A**. HPLC chromatogram; **B**. Experimental mass spectrum (sum of spectra, time window indicated as a grey arrow); **C**. Deconvoluted ions set, including charge state; **D**. Deconvoluted spectrum; **E**. Identified compounds.

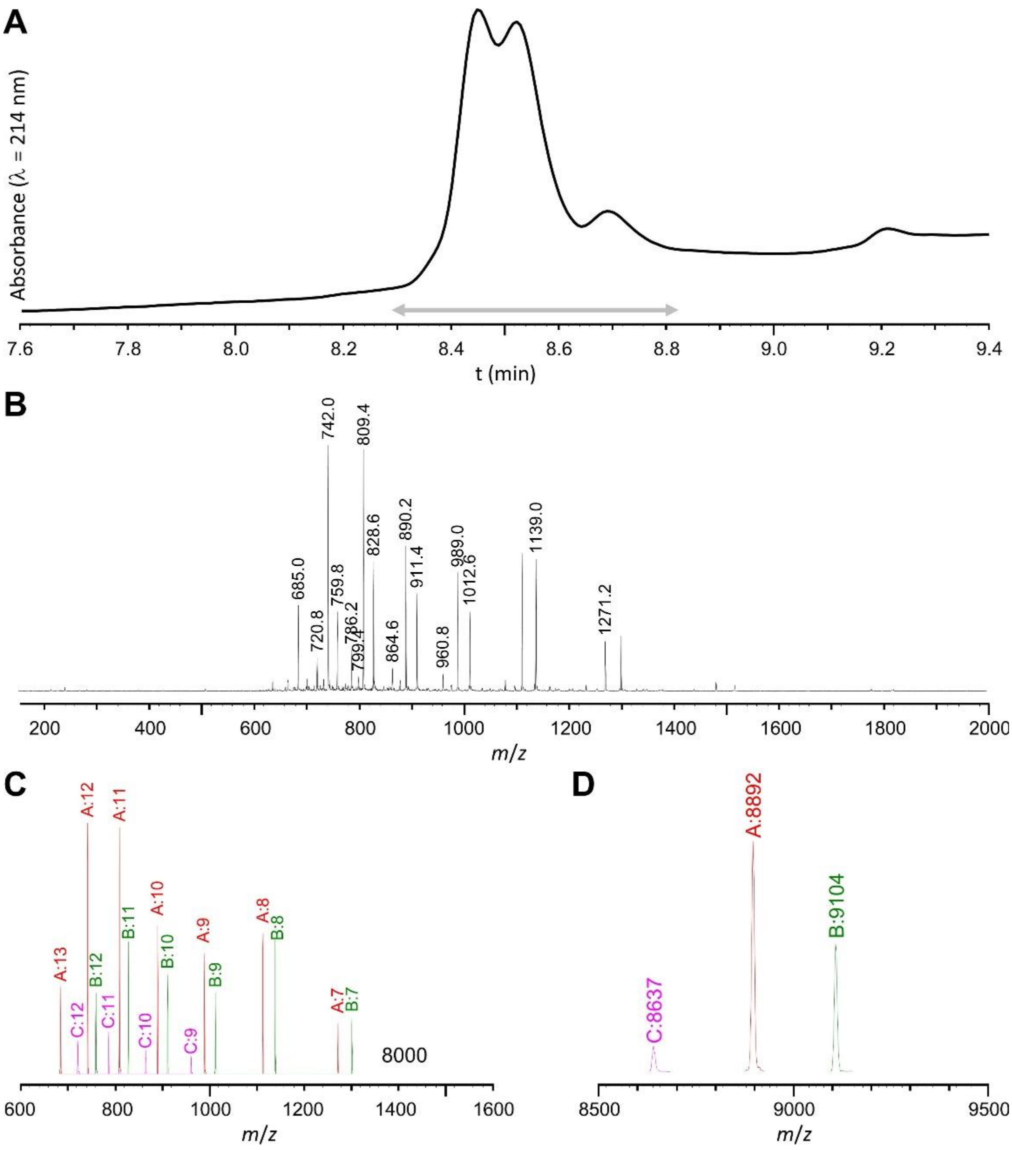

E

Compound	Relative abundance (%)	Detected average mass	Identified product, its theoretical average mass
A	59.9	8892.4 Da	Ub-AMP, 8893.9 Da
B	33.5	9104,3 Da	Ub-ADP-ribose, 9106.0 Da
C	6.6	8637.2 Da	Ub-glycerol, 8638.8 Da

Figure S2 LCMS analysis of NUDT16-mediated cleavage of ubiquitylated ADPr obtained using DTX3L RING-DTC. A. HPLC chromatogram; **B**. Experimental mass spectrum (sum of spectra, time window indicated as a grey arrow); **C**. Deconvoluted ions set, including charge state; **D**. Deconvoluted spectrum; **E**. Identified compounds.

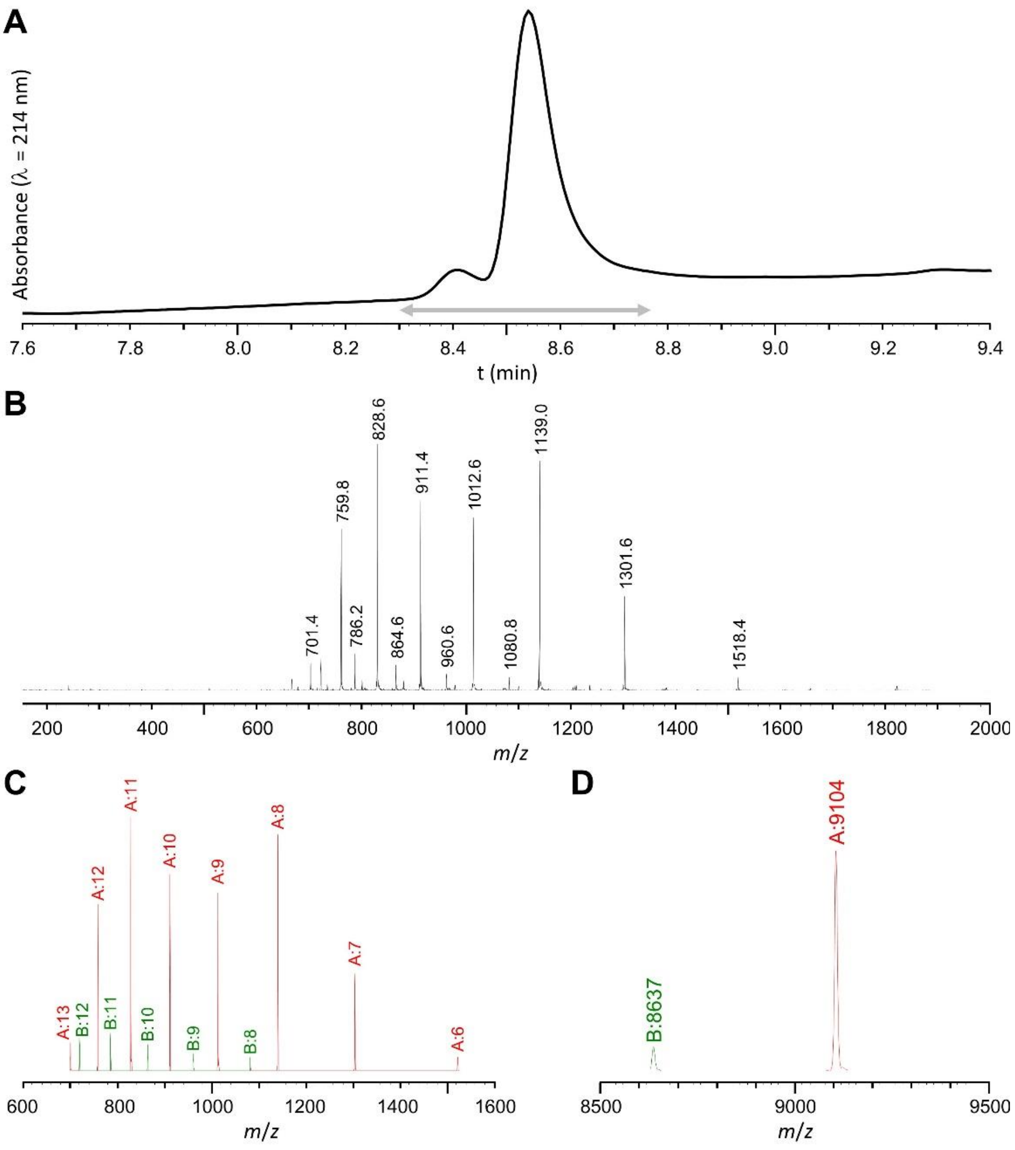

E

Compound	Relative abundance (%)	Detected average mass	Identified product, its theoretical average mass
A	90.1	9104.4 Da	Ub-ADP-ribose, 9106.0 Da
B	9.9	8637.0 Da	Ub-glycerol, 8893.9 Da

Figure S3 LCMS analysis of ADPr ubiquitylation mediated by PARP9:DTX3L. **A**. HPLC chromatogram; **B**. Experimental mass spectrum (sum of spectra, time window indicated as a grey arrow); **C**. Deconvoluted ions set, including charge state; **D**. Deconvoluted spectrum; **E**. Identified compounds.

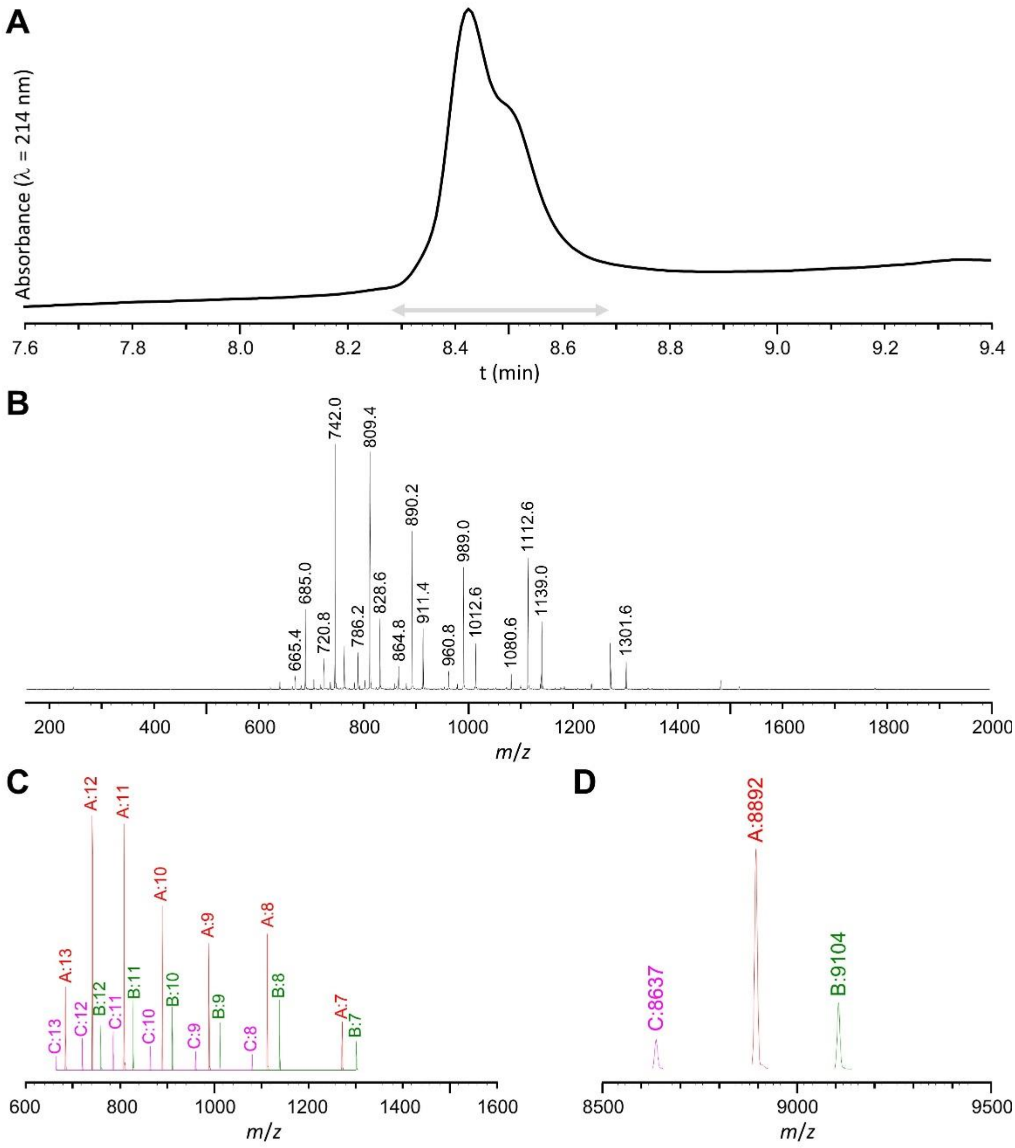

E

Compound	Relative abundance (%)	Detected average mass	Identified product, its theoretical average mass
A	69.5	8892.3 Da	Ub-AMP, 8893.9 Da
B	21.1	9104.3 Da	Ub-ADP-ribose, 9106.0 Da
C	9.4	8637.0 Da	Ub-glycerol, 8638.8 Da

Figure S4 LCMS analysis of NUDT16-mediated cleavage of ubiquitylated ADPr obtained using PARP9:DTX3L. A. HPLC chromatogram; **B**. Experimental mass spectrum (sum of spectra, time window indicated as a grey arrow); **C**. Deconvoluted ions set, including charge state; **D**. Deconvoluted spectrum; **E**. Identified compounds.

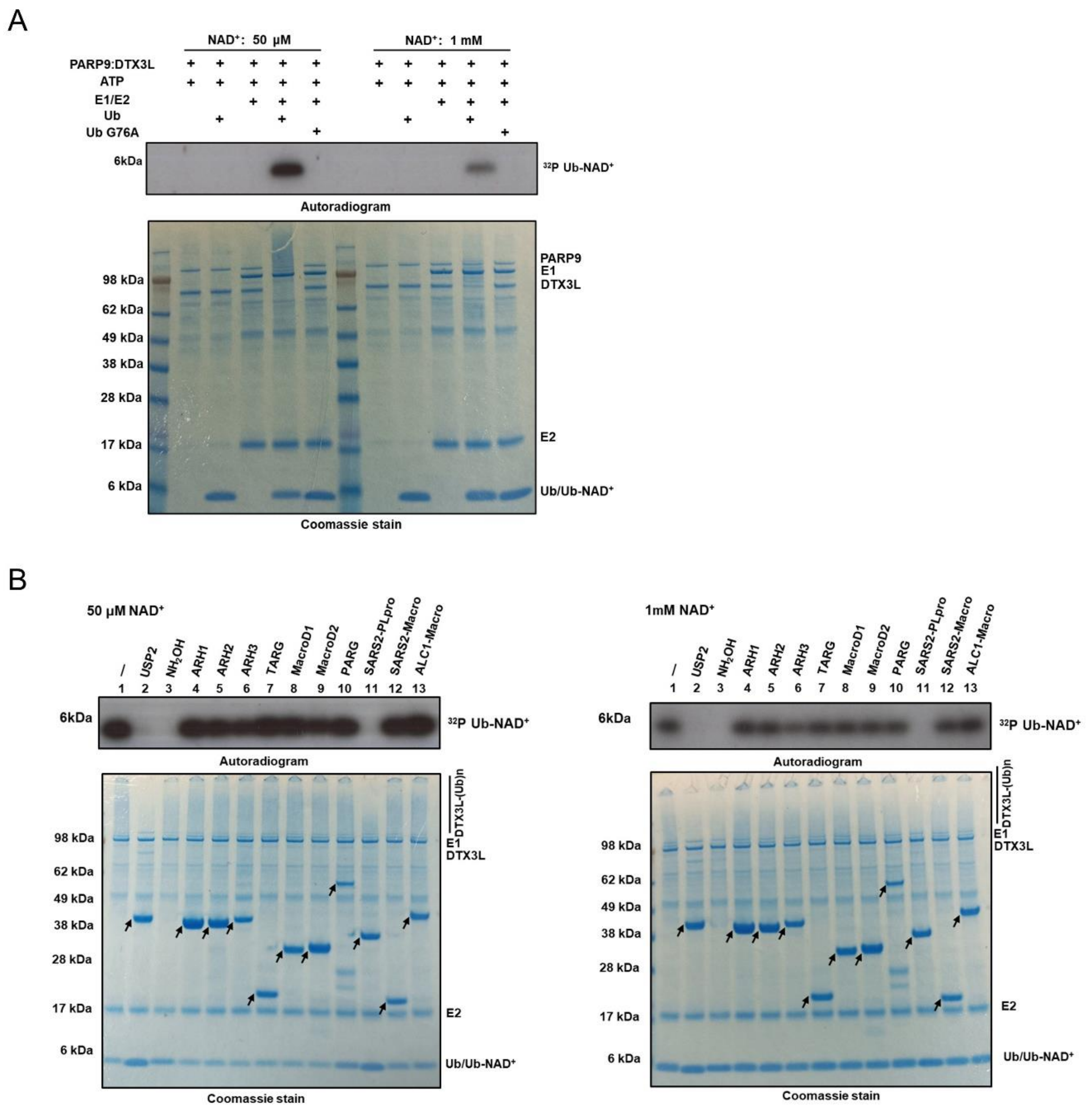

Figure S5. Hydrolase sensitivity of PARP9:DTX3L catalysed Ub-NAD$^+$.

A. Biochemical reconstitution of the Ub-NAD$^+$. ^{32}P Ub-NAD$^+$ was obtained by incubation of PARP9:DTX3L and E1, E2, ATP, Ub, and NAD$^+$ spiked with ^{32}P NAD$^+$. Omitting any of these components blocked the ^{32}P Ub-NAD$^+$ generation. The reactions were analyzed on an SDS-PAGE gel and visualized by Coomassie staining and autoradiography. **B.** Hydrolysis of PARP9:DTX3L catalysed Ub-NAD$^+$. Following NAD$^+$ ubiquitylation reaction performed with PARP9:DTX3L, the indicated ADPr hydrolases, DUBs or NH_2OH were added and further incubated. The samples were analyzed on an SDS-PAGE gel, next visualized by Coomassie staining and autoradiography. The arrows indicate various hydrolases.

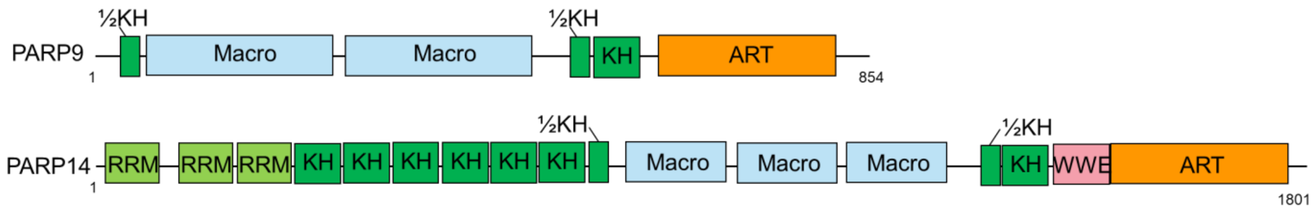

Figure S6. Domain organizations of PARP9 and PARP14. Multiple single-stranded nucleic acids binding domains, such as KH and RRM domains, are present in PARP9 and PARP14.

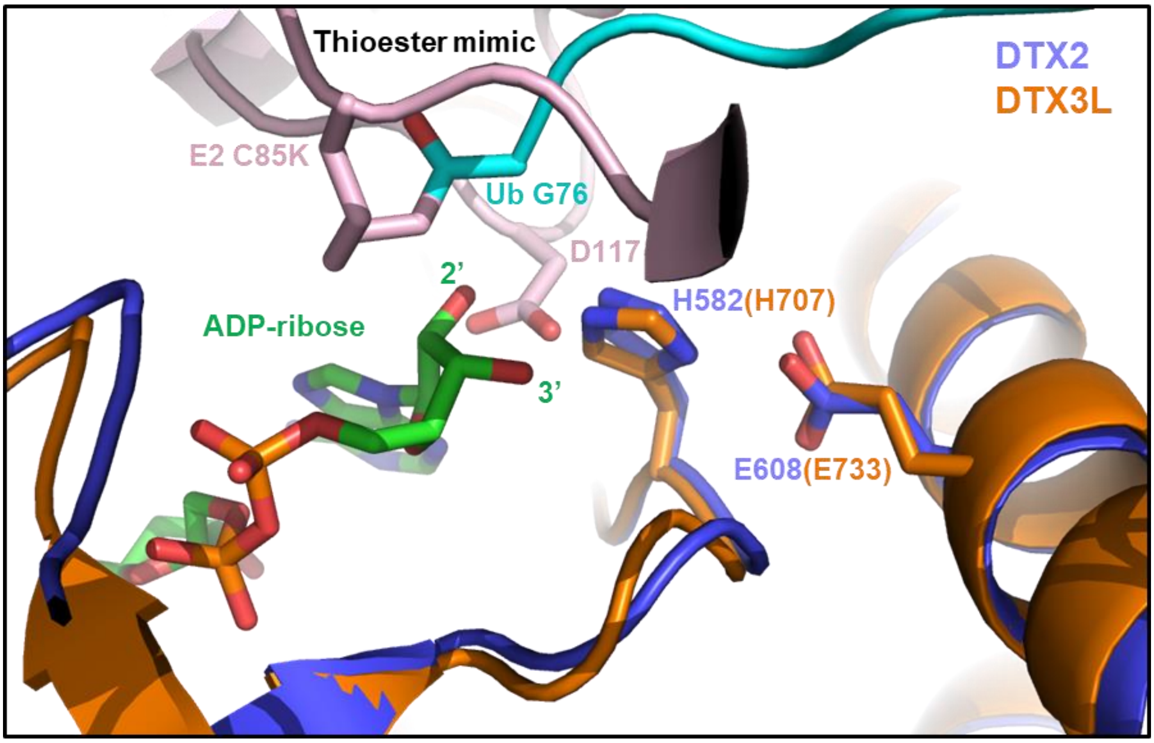

Figure S7 Comparison of the catalytic residues of DTX2 and DTX3L involved in ADP-ribose ubiquitylation. DTX2 RING-DTC: ADP-ribose [Protein Data Bank (PDB): 6Y3J] was aligned with RNF38:UBCH5A~Ub (PDB: 4V3L) on the RING domain (RNF38 is not shown), and then DTX3L (AlphaFold: AF-Q8TDB6-F1) was aligned with DTX2 on the DTC domain. DTX3L's H707 and E733 have similar orientations as DTX2's H582 and E608, which are responsible for deprotonating the 3'-OH of proximal ribose of ADP-ribose to facilitate the ubiquitylation of ADP-ribose.

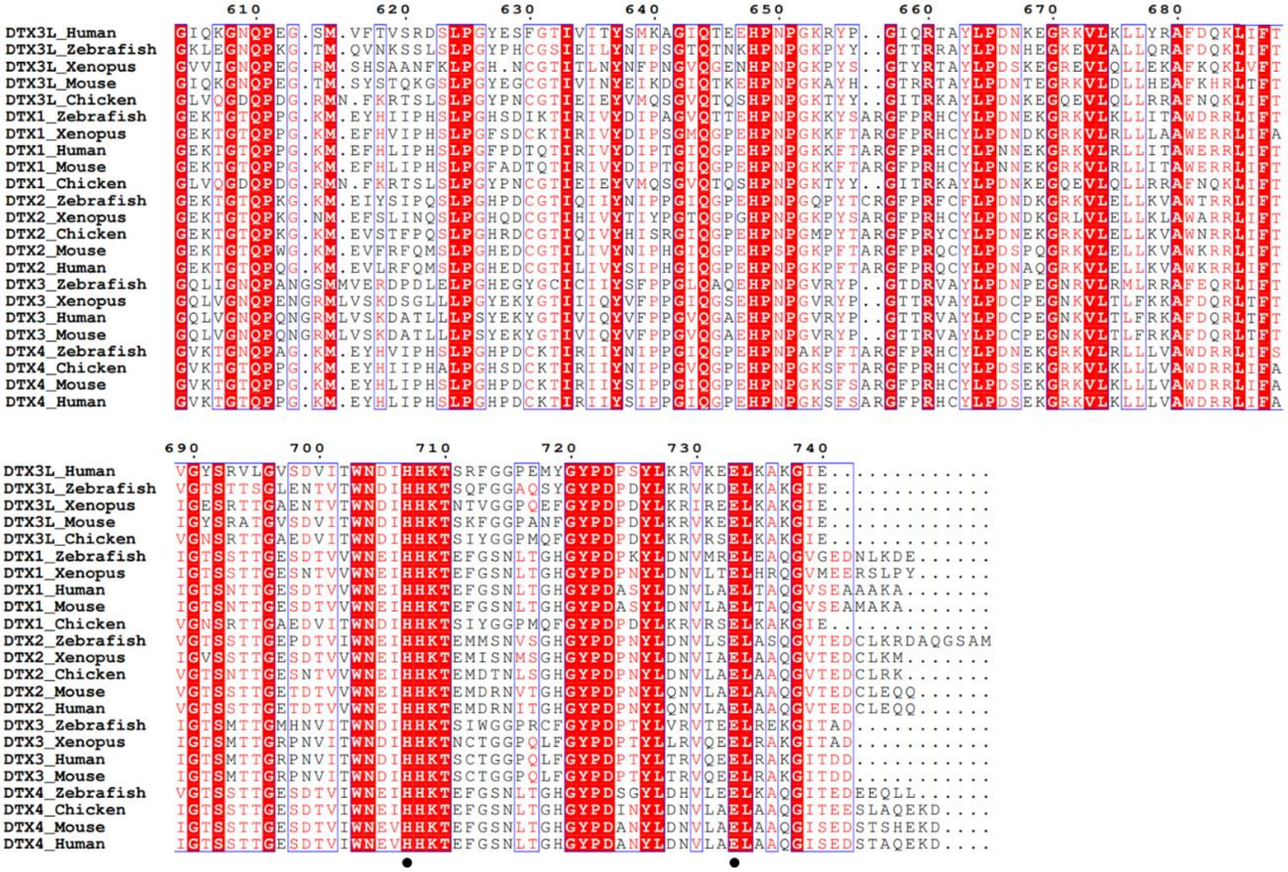

Figure S8 Multiple sequence alignment of the DTC domains from the DELTEX family members. Sequence alignment of DTC domains was generated using Clustal Omega and the figure was prepared using ESPript (http://espript.ibcp.fr). In this alignment, residues that are absolutely conserved and highly conserved are highlighted in dark and light red, respectively. The strictly conserved catalytic residues H707 and E733 (human DTX3L numbering) are marked with solid circles.
